# Genetic Analysis and Functional Study of a Pedigree With Bruck Syndrome Caused by *PLOD2* Variant

**DOI:** 10.3389/fped.2022.878172

**Published:** 2022-05-06

**Authors:** Ruo-li Wang, Dan-dan Ruan, Ya-nan Hu, Yu-mian Gan, Xin-fu Lin, Zhu-ting Fang, Li-sheng Liao, Fa-qiang Tang, Wu-bing He, Jie-wei Luo

**Affiliations:** ^1^Shengli Clinical Medical College, Fujian Provincial Hospital, Fujian Medical University, Fuzhou, China; ^2^Department of Emergency, Fujian Provincial Hospital, Fuzhou, China; ^3^Fujian Trauma Medical Center, Fuzhou, China; ^4^Department of Pediatrics, Fujian Provincial Hospital, Fuzhou, China; ^5^Department of Intervention, Fujian Provincial Hospital, Fuzhou, China; ^6^Department of Hematology, Fujian Provincial Hospital, Fuzhou, China; ^7^Department of Orthopedics, Fujian Provincial Hospital, Fuzhou, China; ^8^Department of Traditional Chinese Medicine, Fujian Provincial Hospital, Fuzhou, China

**Keywords:** Bruck syndrome, *PLOD2* variant, collagen cross-linking, osteogenesis imperfecta, joint contracture

## Abstract

**Background:**

Bruck syndrome (BS) is a rare autosomal recessive inherited osteogenesis imperfecta disease characterized by increased bone fragility and joint contracture. The pathogenic gene of type I BS is *FKBPl0*, whereas that of type II BS is *PLOD2*. No significant difference has been found in the clinical phenotype between the two types of BS. In this study, we performed genetic analysis of a BS pedigree caused by *PLOD2* variant and studied the corresponding cellular function.

**Methods:**

Serum biochemistry, parathyroid hormone (PTH), 25-hydroxyvitamin D [25-(OH) D], osteocalcin, and 24-h urinary calcium levels of a family member with BS was assessed. The genes of the proband were analyzed by second-generation sequencing and exon capture techniques. Sanger sequencing was also performed for the suspected responsible variant of the family member. Wild- and variant-type lentivirus plasmids were constructed by gene cloning and transfected into HEK293T cells. Cell function was verified by real-time quantitative polymerase chain reaction, western blotting, and immunofluorescence detection.

**Results:**

In this pedigree, the proband was found to have a homozygous variant c.1856G > A (p.Arg619His) in exon 17 of *PLOD2* (NM_182943.3). His consanguineous parents and sisters were p.Arg619His heterozygous carriers. The mRNA expression of *PLOD2* in the constructed p.Arg619His variant cells was significantly upregulated, while the expression of PLOD2 and collagen I protein in the cell lysate was significantly downregulated. Immunofluorescence revealed that the wild-type *PLOD2* was mainly located in the cytoplasm, and the expression of the PLOD2 protein after c.1856G > A variant was significantly downregulated, with almost no expression, aligning with the western blot results. The serum sodium, potassium, calcium, phosphorus, magnesium, alkaline phosphatase, PTH, 25-(OH) D, osteocalcin, and 24 h urinary calcium levels of the proband, his parents, and sisters were normal.

**Conclusion:**

Through gene and cell function analyses, *PLOD2* Arg619His missense variant was preliminarily confirmed to cause BS by reducing protein expression.

## Introduction

Bruck syndrome (BS) is a rare osteogenesis imperfecta (OI) disease that mainly manifests as congenital joint contracture, recurrent fragility fracture, scoliosis, and short stature (1). In 1897, Bruck first reported a case of OI with congenital joint contracture, hence the name of the disease (2). OI is a hereditary connective tissue disease characterized by increased bone fragility and disturbance of type I collagen synthesis and metabolism, most of which are of autosomal dominant inheritance (3). BS is an autosomal recessive OI (AR-OI) that can be divided into type 1 BS, which is caused by *FKBPl0* variant, and type 2 BS, which is caused by *PLOD2* variant (4). Currently, there is no known clinical phenotypic difference between *FKBPl0* and *PLOD2* (5).

*PLOD2* is located on chromosome 3q23-q24 and contains 19 exons, encoding lysyl hydroxylase 2 (LH2) (6). Through alternative splicing, *PLOD2* produces two LH2 subtypes with different lengths, namely LH2a (short) and LH2b (long), the former lacking 21 amino acids coded by exon 13A (7). LH2b is mainly distributed in fibrous collagen-rich tissues and is considered to be the main subtype of telopeptide Lys hydroxylation (8). *PLOD2*, *PLOD1*, and *PLOD3* belong to the 2-oxo-glutarate–dependent enzyme family. The proteins they encode are highly homologous, and the overall identity of the protein sequence is 47% (9, 10). PLOD protein has binding sites for cofactor Fe 2 + and L-ascorbic acid, and it also contains 26 amino acids signal peptide and a Prolyl 4-hydroxylase α subunit homology domain (10). *PLOD2* is preferentially expressed in cells with osteogenic activity (11). *PLOD2* changes the activity and stability of LH2, resulting in a serious lack of hydroxylation of the collagen telopeptide lysine residues and a significant reduction in bone type I collagen cross-linking, which ultimately lead to severe abnormalities in bone hardness and structure (12). Although the incidence of AR-OI is less than 10%, the clinical manifestations induced are more serious than those induced by dominant inheritance (13).

Due to the limited number of patients with BS and the obvious overlap between BS and OI in clinical phenotypes, genetic analysis of BS and the functional study of variant genes have always been difficult. Our study identified a family with BS caused by a *PLOD2* variant through sequencing. To identify the function of the variant gene at the molecular and cellular levels, wild and variant *PLOD2* were inserted into a lentiviral plasmid vector and transfected into HEK293T cells. The expression and location of the PLOD2 protein in HEK293T cells were detected by immunofluorescence, and the expression of *PLOD2* mRNA and protein in different transfection groups was detected by real-time quantitative polymerase chain reaction (RT-qPCR) and western blotting.

## Materials and Methods

### Case Presentation

**FIGURE 1:**
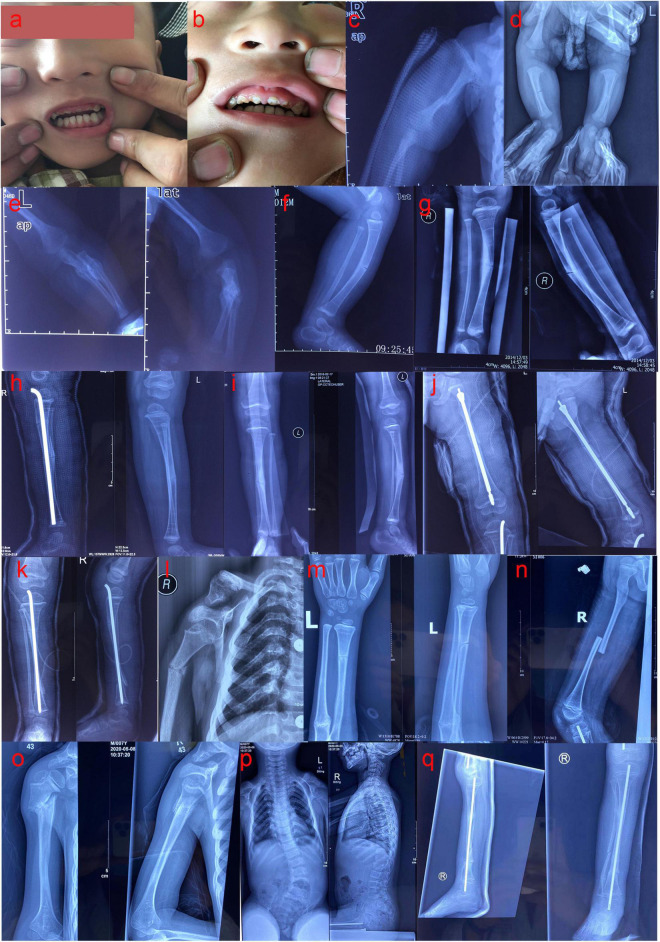
**(a,b)** The enamel of the left and right central incisors, lateral incisors, and canines of the proband’s maxilla were evidently exfoliated, showing a brownish-yellow appearance; the abovementioned teeth were severely worn, and some were close to the gingival margin. **(c)** Anteroposterior radiograph (AP) of the right humerus at 1 month after birth: fracture of the right humerus. **(d)** AP of both lower limbs at 1 month after birth: fracture of the right tibia. **(e)** The anteroposterior and lateral radiographs of the left ulna and radius at 1 month after birth: fracture of the left ulna and radius. **(f)** Lateral radiographs of the left tibia and fibula at the age of 1 year: left tibia fracture. **(g)** The anteroposterior and lateral radiographs of the right tibia and fibula at 1 year and 11 months: fracture of the right tibia. **(h)** Bilateral tibia and fibula lateral radiographs at 2 years old: the right upper tibia fractured when the right middle tibia fracture was fixed with intramedullary rod internal fixation, and combined with the left tibia fracture. **(i)** The anteroposterior and lateral radiographs of the left tibia and fibula at 3 years and 1 month: fracture of the left tibia. **(j)** The anteroposterior and lateral radiographs of the left femur at 3 years and 2 months: multiple fractures of the left femur. **(k)** The anteroposterior and lateral radiographs of the right tibia and fibula at 3 years and 3 months: multiple fractures of the right tibia. **(l)** AP of the right humerus at 5 years and 7 months: right humerus fracture. **(m)** The anteroposterior and lateral radiographs of the left ulna and radius at 6 years and 8 months: fracture of the left ulna and radius. **(n)** Lateral radiograph of the right femur at 6 years and 9 months: fracture of the right femur. **(o)** The anteroposterior and lateral radiographs of the right humerus at 7 years and 4 months: fracture of the right humerus. **(p)** The anteroposterior and lateral radiographs of the whole spine showed severe scoliosis at 7 years and 4 months. **(q)** The anteroposterior and lateral radiographs of the right tibia and fibula at 7 years and 10 months: multiple fractures of the right tibia and fibula.

The proband (II 2) (male, 8 years old, and of Han nationality) complained of recurrent limb fractures for 8 years. The proband is the second child of a pair of consanguineous parents (I1 and I2). He was born with a normal Apgar score, weighing 3,000 g and a height of 49.1 cm. The indexes of his sister (II 1) were similar to those of children of the same age. The proband did not have hyperthyroidism, rheumatic heart disease, diabetes, and hypertension, and did not have a history of hepatitis, tuberculosis, and cardiovascular disease. Family members had no similar medical history and denied any family genetic history. The proband had short stature (approximately 113 cm in height), scoliosis, knee and ankle contracture, and foot varus deformity in both lower limbs. In addition, the enamel of the left and right central incisors, lateral incisors, and canines of the proband’s maxilla were evidently exfoliated, displaying a brownish-yellow appearance. The abovementioned teeth were severely worn, and some were close to the gingival margin (Figures 1a,b). His vision and hearing were normal, with no visual field defect, no pterygium, and no obvious abnormalities in the rest of the physical examination. To complete the examination, serum biochemistry, parathyroid hormone (PTH), 25-hydroxyvitamin D [25-(OH) D], osteocalcin, and 24-h urinary calcium levels were determined. This study was approved by the Ethics Committee of Fujian Provincial Hospital, and all the family members participating in this study signed informed consent forms.

### Methods

#### Extraction of Genomic DNA From Peripheral Blood

The peripheral blood of family members was collected, and the QIAGEN DNA Blood Mini Kit (Cat#51106, QIAGEN Co., Ltd.) was used to extract the genomic DNA of the proband and other family members. The concentration and purity of the extracted DNA were detected using a NanoDrop instrument and Qubit fluorometer, and the degree of DNA fragmentation was detected by agarose gel electrophoresis.

#### Candidate Gene Location and Variant Gene Screening Strategy

The TargetSeq^®^ liquid phase probe hybrid capture technology independently developed by iGeneTech^®^ (Beijing, China) was used to establish a genomic DNA library and capture the promoter and exon regions (16.06 Mbp) of 5,081 genes associated with genetic diseases. The Illumina X10 or NovaSeq6000 platform was used for paired-end 150 bp sequencing. The captured target genes included *COL1A1*, *COL1A2*, *FKBP10*, *PLOD2*, *CRTAP*, *SERPINH1*, *BMP1*, and *WNT1*. The sequenced reads were processed by Burrows-Wheeler Aligner and compared with the reference sequence of hg19. According to the bam results of alignment with genome reference sequences, the single nucleotide variants and indels in sequencing data were analyzed using ANNOVAR, samtools, GATK, and other software; compared with the human genome information in dbSNP, 1,000 g, HGMD, and ESP6500 databases according to the American College of Medical Genetics and Genomics guidelines; and screened for suspicious variants. The source of variants was then annotated. Polymorphism phenotyping (PolyPhen-2),^1^ Sorting Intolerant from Tolerant (SIFT),^2^ and Mutation Taster^3^ were employed to predict the variant function. The primer design software premier 5.0 was used to design the specific primers of the upstream and downstream positions of the sequence containing the target variant site, and the target region was amplified. Sanger sequencing was performed on the ABI 3,500 Dx platform to verify the next-generation sequencing (NGS) results. The amplified fragment length of the target sequence of c.1856G > A (p.Arg619His) of the variant point *PLOD2* (NM_182943.3) was 545 bp. The primers used were F: AAAGCAAAGTAAGCCAGTGGATTT and R: GTGAGGGCTTAGGAAGTCAATTTTA. The annealing temperature was maintained at 60°C. The primers were synthesized by Synbio Technologies (Suzhou, China).

#### Construction and Identification of *PLOD2* Wild Type and p.Arg619His Variant Plasmid

The following method was employed for plasmid synthesis. pCDH-CMV-MCS-EF1- copGFP-T2A-puro was used as the expression vector to synthesize *PLOD2*. The insert size was 2,294 bp. *Xba*I and *Not*I were selected as the double restriction enzymes (see Supplementary Material 1 for the sequence). The wild type (WT) plasmid pCDH-CMV-hPLOD2-EF1- copGFP-T2A-Puro (see Supplementary Material 1 for the sequence) and the variant plasmid pCDH-CMV-hPLOD2 mut-EF1-copGFP-T2A-Puro (see Supplementary Material 2 for the sequence) were constructed, both with *Xba*I and *Not*I restriction enzyme sites. The c.1856G > A variant site of *PLOD2* was introduced into the variant plasmid. Target genes were amplified and sequenced. *PLOD2* (WT), *PLOD2* (c.1856G > A) gene cloning, and related PCR primers synthesis were performed by Wuhan Gene Create Biological Engineering Co., Ltd. (Wuhan, China).

#### Cell Transfection

HEK293T cells were collected by trypsin digestion, and the cells were placed on a 10-cm cell culture dish with a density of 1–2 × 10^7^ cells/plate using an appropriate complete culture medium (the total area occupied by the cells after adhesion was 80–90% of the culture dish area). According to the condition of cell adhesion, the cells were incubated at 37°C incubator containing 5% CO_2_ for 8–24 h, and transient transfection was initiated after the cells were adhered completely. TurboFect-DNA Mix was prepared according to the instructions of TurboFect (R0531, Thermo, GER). The DNA plasmid (10 μg/*PLOD2* WT or variant or control plasmid + 5 μg green fluorescent protein (GFP) control plasmid) and 30 μL TurboFect in 1,000 μL Opti-Medium were gently mixed and incubated at room temperature for 15 min. TurboFect-DNA Mix was added to the culture dishes. After 12 h, the complete medium was changed, and the cells were cultured for 48 h. The cells appeared to be in good condition based on microscopy analysis, and the culture medium was collected for further treatment.

#### Determination of *PLOD2* mRNA Expression Level by Real-Time Quantitative Polymerase Chain Reaction

The instructions of the TRIPURE ISOLATION REAGENT kit (11667165001, Roche, Switzerland) were followed to extract HEK293T total RNA, and the difference in *PLOD2* transcription level was detected by reverse transcription and RT-qPCR. According to the instructions of HiFiScript (CW2020M, CWBIO, Beijing, CHN), the first chain of cDNA was synthesized, and the reaction system comprised 2.5 mM dNTP Mix, 4 μL; Primer Mix, 2 μL (primer in Table 1); RNA Template, 7 μL; 5 × RT Buffer, 4 μL; DTT, 0.1 M, 2 μL; HiFiScript, 200 U/μL, 1 μL; and RNase-Free Water to a final volume of 20 μL. The liquid was mixed by vortex concussion and centrifuged briefly so that the solution on the wall of the pipe was collected to the bottom of the tube. The samples were incubated at 42°C for 50 min and then at 85°C for 5 min. The cDNA obtained by reverse transcription was diluted 20 times for RT-qPCR, and RT-qPCR was performed for 40 cycles in a Roche LightCycler 480 (Roche, Beijing, China).

**TABLE 1:** Primers for RT-qPCR.

hPLOD2 qRT F	GGGAGTTCATTGCACCAGTT
hPLOD2 qRT R	CATGAAGCTCCAGCCTTTTC
hGAPDH F	AGAAGGCTGGGGCTCATTTG
hGAPDH R	AGGGGCCATCCACAGTCTTC
CopGFP qRT F	AGGACAGCGTGATCTTCACC
CopGFP qRT R	CTTGAAGTGCATGTGGCTGT

#### Determination of PLOD2 Protein Expression by Western Blot

HEK293T cells were cultured and lysed. Thereafter, the total protein of HEK293T cells was extracted and the expression of the PLOD2 protein was detected. After the electrophoresis device was installed, the protein sample and Maker were added into the gel pore according to the desired order, and then wet transferred onto the polyvinylidene fluoride (PVDF) membrane. After protein transfer, the membrane was rinsed with Tris buffered saline Tween (TBST) and sealed with 5% bovine serum albumin (BSA) blocking solution for 1 h at room temperature. After sealing, the PVDF membrane was rinsed once with TBST for 5 min. The primary antibody was diluted with 5% BSA to an appropriate concentration (Flag antibody, 1: 1,000; tubulin antibody, 1: 2,000; Collagen I antibody, 1: 1,000; GFP antibody, 1: 1,000), added to the PVDF membrane and reacted at 37°C for 1 h or 4°C overnight. Thereafter, the membrane was washed three times, and the secondary antibody labeled with horseradish peroxidase was diluted with 5% BSA (dilution ratio 1: 2,000), and reacted at room temperature for 1 h. Finally, the PVDF membrane was washed five times with TBST and once with ddH2O. The exposure substrate was added for development.

#### Immunofluorescence Detection of Cell Localization

After being fixed, permeated, and blocked, the transfected HEK293T cells were added with FLAG first antibody (1:1,000 dilution) and incubated overnight at 4°C. The cells were rinsed three times with phosphate buffered saline and 0.05% TWEEN^®^ 20 (PBST), incubated with fluorescent secondary antibody (diluted ratio 1: 500) at room temperature for 2 h, rinsed three times with PBST, stained with 4, 6-diamidino-2-phenylindole, and incubated at room temperature for 10 min. Thereafter, the cells were rinsed twice with 1 × PBS for 3 min each time, sealed with 50% glycerol, and photographed using a laser confocal microscope (Nikon A1, JPN).

#### Statistics

The experimental data were statistically analyzed using GraphPad Prism 6.02. One-way analysis of variance was used to compare three groups, and the least significant difference test was used for comparison between two groups. The mean value was expressed as mean ± standard error of the mean, and *P* < 0.05 was considered statistically significant.

## Results

### Clinical Phenotype of the Proband

**FIGURE 2:**
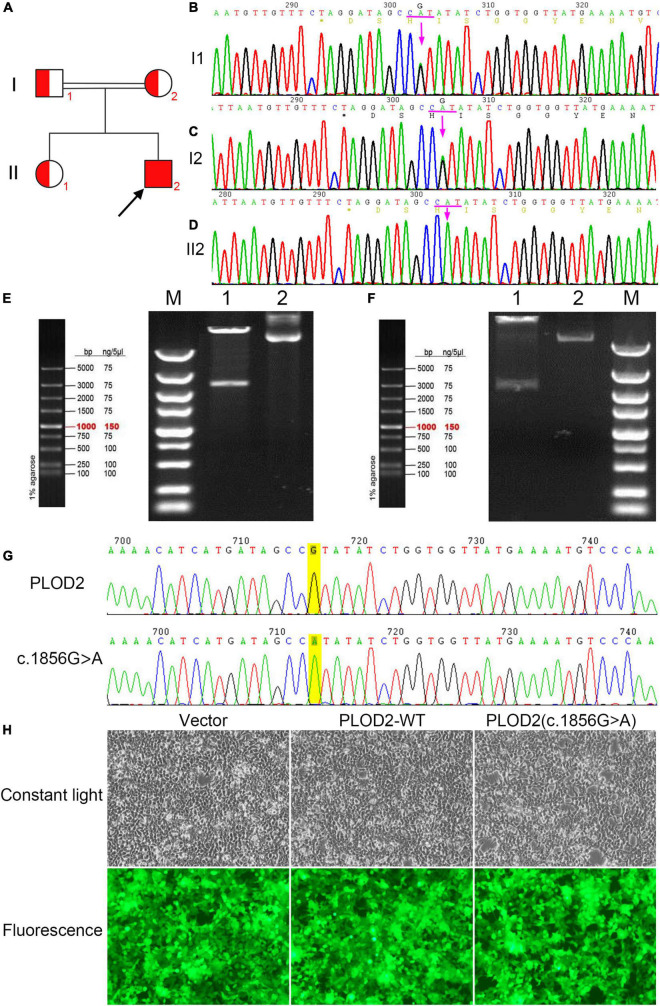
**(A)** Bruck syndrome (BS) pedigree map; the proband (arrow) has a homozygous variant; red indicates carrying the c.1856G > A (p.Arg619His) variant of *PLOD2* gene. **(B,C)** A heterozygous variant of c.1856G > A (p.Arg619His) in exon 17 of *PLOD2.* **(D)** A homozygous variant of c.1856G > A (p.Arg619His) in exon 17 of *PLOD2.* **(E)** The electrophoretic map of *PLOD2* (c.1856G > A) clone plasmid digested by *Xba*I and *Not*I. Lane M, DNA Marker; Lane 1, plasmid digested by *Xba*I and *Not*I; and Lane 2, plasmid DNA. **(F)** The electrophoretic map of *PLOD2* wild type clone plasmid digested by *Xba*I and *Not*I. Lane M, DNA Marker; Lane 1, plasmid digested by *Xba*I and *Not*I; Lane 2, plasmid DNA. **(G)** Sanger sequencing verification diagram of *PLOD2* wild type and c.1856G > A clone plasmid. **(H)** The cell growth diagram after cell transfection showed that there was no significant difference in GFP transfection efficiency among the groups.

One month after birth, the proband spontaneously developed fractures of the right humerus, tibia, and left ulna and radius without obvious induction (Figures 1c–e). Accordingly, he was treated with plaster external fixation. From the age of 2 months onward, he was treated with intravenous infusion of pamidronate every 6 months, to increase bone mineral density. At 1 year-old, the proband suffered left tibial fracture (Figure 1f) and left ulnar fracture without obvious external force. The fracture of the left femur occurred at 1 year and 2 months, and the affected limb recovered well after skin traction and plaster external fixation of the left lower limb. The right middle tibia fracture occurred again at 1 year and 11 months (Figure 1g), and the upper tibia fracture occurred when the right middle tibia fracture was fixed with intramedullary rod internal fixation at the age of 2 years; the left tibia fracture occurred at the same time (Figure 1h). During this period, the proband regularly received intravenous bisphosphate treatment to increase bone mineral density. However, the left tibia fracture occurred again at the age of 3 years and 1 month (Figure 1i), and multiple fractures of the left femur and right tibia occurred at the ages of 3 years and 2 months, and 3 years and 3 months, respectively (Figures 1j,k); these fractures were treated with intramedullary rod internal fixation. At the age of 5 years and 7 months, and 6 years and 8 months, the proband had fractures of the right humerus and left ulna and radius, respectively (Figures 1l,m). The right femur fracture occurred at the age of 6 years and 9 months (Figure 1n), and was treated with skin traction and plaster external fixation of the right lower limb. At 7 years and 4 months, the proband had a fracture of the right humerus again (Figure 1o), and the anteroposterior and lateral radiographs of the whole spine revealed severe scoliosis deformity (Figure 1p). At 7 years and 10 months, the proband had multiple fractures of the right tibia and fibula (Figure 1q) and was treated with intramedullary rod internal fixation of the right lower limb. The results of laboratory examination showed that serum sodium, potassium, calcium, phosphorus, magnesium, alkaline phosphatase, PTH, 25-(OH) D, and osteocalcin were within the normal range, and 24 h urinary calcium levels were also generally normal (Table 2). The biochemical indices of his parents and sister were normal (Table 2). Based on the results of the pedigree investigation, a genetic family tree was plotted (Figure 2A).

**TABLE 2:** Clinical data of proband’s family.

Item	Proband (II 2)	Father (I 1)	Mother (I 2)	Sister (II 1)
Gender	Male	Male	Female	Female
Age, years	10	40	40	14
Height, cm	117	178	150	164
Weight, kg	17	80	57	45
Recurrent fragility fracture	+	−	−	−
Congenital joint contracture	+	−	−	−
Scoliosis	+	−	−	−
Dentinogenesis imperfecta	+	−	−	−
Aspartate aminotransferase, U/L	18	28	23	20
Gamma-glutamyl transferase, U/L	22	33	29	30
Alkaline phosphatase, U/L	125	111	115	108
Lactic dehydrogenase, U/L	202	189	226	195
Serum sodium, mmol/L	141	139	142	140
Serum potassium, mmol/L	4.1	4.5	4.7	4.0
Serum calcium, mmol/L	2.23	2.35	2.21	2.36
Serum phosphorus, mmol/L	1.17	0.97	1.15	1.26
Serum magnesium, mmol/L	0.86	0.92	0.85	0.95
Parathormone, Pg/mL	46.93	55.41	43.21	42.57
25-Hydroxyvitamin D, ng/mL	25.81	22.39	28.76	26.56
Osteocalcin, μg/L	25.11	29.87	26.31	30.17
24 h Urinary calcium, mmol/day	4.8	5.3	4.3	5.1

*Normal reference values: aspartate aminotransferase, 13–35 U/L; gamma-glutamyl transferase, 7–45 U/L; alkaline phosphatase, 50–135 U/L; lactic dehydrogenase, 120-250 U/L; serum sodium, 137–147 mmol/L; serum potassium, 3.5–5.3 mmol/L; serum calcium, 2.11–2.52 mmol/L; serum phosphorus, 0.85–1.51 mmol/L; serum magnesium, 0.75–1.02 mmol/L; parathormone, 15–88 Pg/mL; 25-hydroxyvitamin D, 20–32 ng/mL; osteocalcin, 11–43 μg/L; 24 h urinary calcium, 2.5–7.5 mmol/day.*

### Screening for Variant Genes

Through NGS and exon capture technology, the proband (II2) was found to have a homozygous variant c.1856G > A (p.Arg619His) in exon 17 of *PLOD2* (NM_182943.3). This variant transformed CGT into CAT and led to a change from arginine (Arg) to histidine (His) at position 619 of the PLOD2 protein. Both his parents and sister carried a heterozygous variant of p.Arg619His (Figures 2B–D).

### Cloning of *PLOD2* Wild Type and p.Arg619His Variant

The cloning vectors and eukaryotic expression vectors of *PLOD2*—WT and *PLOD2*/p.Arg619His were successfully constructed. Fragments of variant *PLOD2*/p.Arg619His and WT *PLOD2* digested by *Xba*I and *Not*I were approximately of 3,000 bp (Figures 2E,F), aligning with the design. The constructed vectors were verified by sequencing (Figure 2G) and successfully transfected into HEK293T cells (Figure 2H).

### Localization of PLOD2 Wild Type and p.Arg619His Variants in Cells

**FIGURE 3:**
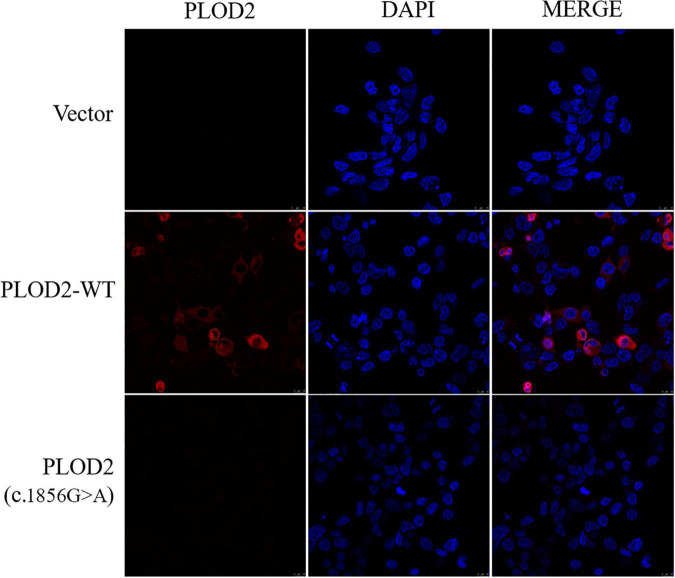
Immunofluorescence was used to detect the localization of PLOD2 protein carrying empty type (Vector), wild type (*PLOD2*-WT), and *PLOD2* p.Arg619His (c.1856G > A) variant in HEK293T cells. After c.1856G > A variant, the expression of PLOD2 protein was significantly downregulated.

The expression and localization of WT and variant PLOD2 proteins were detected by immunofluorescence (Figure 3). Based on the result, WT PLOD2 was mainly located in the cytoplasm. However, after c.1856G > A variant, the expression of the PLOD2 protein was significantly downregulated and showed almost no expression.

### Expression of PLOD2 Wild Type and p.Arg619His Variant in HEK293T Cells

**FIGURE 4:**
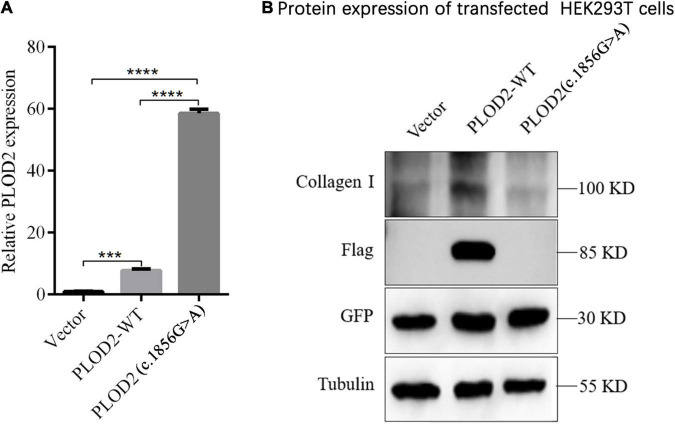
Expression analysis of *PLOD2* in HEK293T cells. **(A)** RT-qPCR detection showed that c.1856G > A variant caused significant overexpression of *PLOD2* gene compared with the empty type (Vector) and wild type (*PLOD2*-WT) groups, ***p < 0.001, ****p < 0.0001. **(B)** Using anti-Flag antibody against Flag-labeled PLOD2, the expression of Flag-PLOD2 and Collagen I protein in Vector, PLOD2-WT, and *PLOD2*- c.1856G > A in HEK293T cell lysate was detected by Western Blot.

The expression of PLOD2, external reference GFP, and internal reference GAPDH was detected by RT-qPCR. When there was no significant difference in GFP transfection efficiency among groups, GFP was used as an external reference to compare the differences of *PLOD2* mRNA between groups (Figure 4A). According to the results, *PLOD2* mRNA expression was significantly upregulated after c.1856G > A variant (*p* < 0.05). The expression of the PLOD2 protein in the cell lysates was detected by western blotting (Figure 4B). The results showed that the expression levels of PLOD2 and Collagen I protein in the cell lysate were significantly downregulated after c.1856G > A variant (*p* < 0.05).

### Prediction and Analysis of Protein Bioinformatics

**FIGURE 5:**
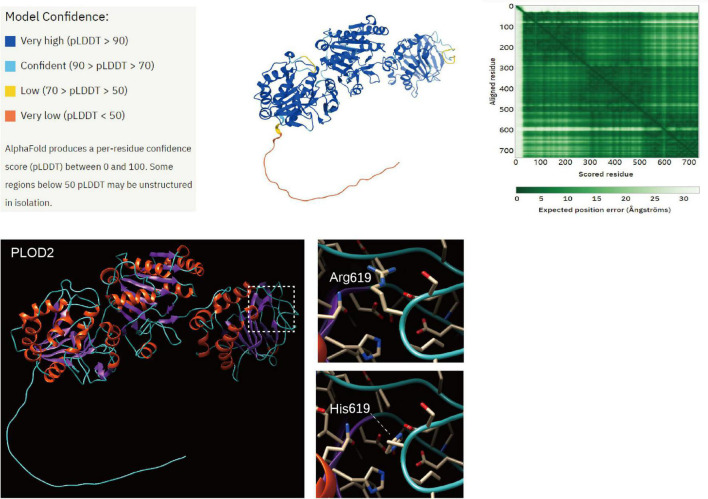
Structure prediction of PLOD2 protein variant. The tertiary structure of the PLOD2 protein was predicted by AlphaFold (https://alphafold.ebi.ac.uk/entry/O00469). Model confidence was ranked by four levels and colored in a tertiary structure. The heatmap shows the confidence in the PLOD2 protein. The first 30 amino acids of the N-terminal of the PLOD2 protein in this model had little accuracy of model confidence, while the other region of PLOD2 protein has desirable confidence. We loaded this predicted model in Chimera 1.15, and the structural change of PLOD2 influenced by an R > H substitution in position 619 (LH2b) of the protein was exhibited and the variant site was labeled. AlphaFold was used to predict the structural changes of p.Arg619His variant, and it was found that this variant may lead to wrong protein folding.

The tertiary structure of the PLOD2 protein was predicted by AlphaFold.^4^ Model confidence was ranked by four levels and colored in a tertiary structure. The heatmap shows the confidence in the PLOD2 protein. The first 30 amino acids of the N-terminal of the PLOD2 protein in this model had little accuracy of model confidence, while the other region of PLOD2 protein has desirable confidence. We loaded this predicted model in Chimera 1.15, and the structural change of PLOD2 influenced by an R > H substitution in position 619 (LH2b) of the protein was exhibited and the variant site was labeled (Figure 5). In our identified variants of patients with Bruck syndrome, His substituted for Arg at residue 619. The sequence fragment of this amino acid is highly homologous in the three human LH isoenzymes and highly conserved in different species. Therefore, this region is considered to be an important functional domain of LH2. AlphaFold was used to predict the structural changes of p.Arg619His variant, and it was found that this variant may lead to wrong protein folding. SIFT online software (see text footnote 2) predicted that this missense mutation might cause protein damage.

## Discussion

BS is an autosomal recessive inherited OI disease caused by a type I collagen cross-linking defect (14). The disease has obvious clinical phenotypic heterogeneity and genetic heterogeneity (6, 15). In bone tissue, the synthesis and degradation of bone collagen are closely related to the elasticity and toughness of bone. *PLOD2* variant has a significant effect on bone strength by causing abnormal cross-linking of bone collagen, leading to type 2 BS (7). Compared with type 1 BS caused by *FKBP10* variant, type 2 BS caused by *PLOD2* variant is more rare. Further, as there is no obvious phenotypic difference between the two types, genetic data are mainly used for classification (16). Until 12/12/2021, there are 12 types of *PLOD2* variants in the HGMD database,^5^ including 8 missense/non-sense, 2 splitting, 1 small deletions, and 1 small insertions variants. The main features of BS include bone fragility of the OI and joint contracture (17, 18). Unlike other OI diseases, patients with BS may not have blue sclera, dentinogenesis imperfecta, or hearing impairment, and their intellectual development may not be affected (19). Interestingly, the proband in this study had manifestations of dentinogenesis imperfecta. Patients with BS may have deformed feet and congenital joint movement limitations and are more likely to develop severe scoliosis (20). Early animal model studies have shown that a change in type I collagen cross-linking might lead to an increase in bone fragility or the occurrence of OI (7).

Type I collagen is the main protein of human bone, dentin, and skin, and accounts for approximately 95% of the bone collagen content (21). Abnormal metabolism can lead to a decrease in bone strength and increase in fracture risk. Type I collagen is a triple helix structure formed by two pre-α1 chains and one pre-α2 chain intertwined with each other, and the center of each pre-α chain consists of more than 300 continuously repeated Gly-X-Y (glycine-proline-hydroxyproline) sequences, flanked by the propeptide structures of the amino terminal and carboxyl terminal (21, 22). The triple helix chains synthesize procollagen molecules in the endoplasmic reticulum and carry out extensive post-translational modifications (23). Subsequently, the Golgi apparatus transports procollagen to the extracellular matrix, in which the amino terminal and carboxyl terminal propeptides are removed by a specific peptidase, and the procollagen molecules are further processed and aggregated to form collagen fibers (14, 24). In collagen fibers, molecules arranged in parallel are longitudinally staggered with each other and stabilized by covalent cross-linking (25).

Cross-linking between collagen molecules is an important post-translational modification process that can affect the mechanical stability and tensile resistance of collagen fibers (26). Depending on whether the residue of telopeptide is lysine (Lys) or hydroxylysine (Hyl), the formation of collagen cross-linking can be divided into two pathways: the allysine and hydroxyallysine routes (27). The stability of type I collagen in bone is mainly derived from the cross-linking of the hydroxyallysine route, and the hydroxylation state of telopeptide Lys residues is very important for the stability of cross-linking (27, 28). In the bone tissue of patients with BS, the level of type I collagen cross-linked by the hydroxyallysine route is decreased (14).

The hydroxylation of telopeptide Lys residues of type I collagen in patients with BS is obviously insufficient, while the hydroxylation of triple helix Lys residues is normal, which is related to the decrease in the activity of telopeptide Lysyl hydroxylase (LH) (27). LH2 is the key enzyme for the formation of stable cross-linking of collagen fibers in the extracellular matrix and is also the only member of the LH family that can hydroxylate the telopeptide Lys residues of type I collagen to hydroxylysine (8, 25, 29). In the late stage of osteoblast differentiation *in vitro*, the expression level of the *PLOD2* gene encoding LH was significantly increased, which was consistent with the increase in telopeptide Lys hydroxylation of type I collagen (11). Such a finding further indicates that the expression of *PLOD2* is related to the hydroxylation of the type I collagen telopeptide Lys. In addition, the FKBP65 protein encoded by *FKBP10* is a molecular chaperone involved in the folding of type I procollagen, which can affect the secretion of type I collagen (30–32). FKBP65 exerts the activity of peptidyl-prolyl cis-transisomerase (PPIase), which can catalyze the conversion of cis-trans isomers of the peptidyl-proline bonds in proteins containing proline (33, 34). This transformation is a rate-limiting step in the folding of newborn proteins. The PPIase activity of FKBP65 can also affect the activity and stability of LH2, thereby participating in the regulation of collagen cross-linking (17, 35). This indirectly explains the similarity of clinical phenotypes between type 1 and type 2 BSs.

In a summary article on 16 different proband families with typical BS phenotypes from different countries and races, at least 17 different *PLOD2* variants were mentioned (36). Most of these variants were homozygous variants of *PLOD2*, and a few carried compound heterozygous variants (36). LH peptides have two domains, the N-terminal and C-terminal, in which the C-terminal domain contains important catalytic residues of LH activity (20). Among the *PLOD2* variants found to date, most are located between exons 12–19 and show obvious C-terminal aggregation (37, 38). As the triple helix of the collagen molecule was assembled from the C-terminal to the N-terminal, a C-terminal variant could lead to more serious collagen structural instability and clinical phenotype (39). LH2b consists of 758 amino acids, including an additional exon 13A of 63 bp (40). The exon encodes 21 amino acids and has different expression levels in different tissues (40). In 2004, a boy who had congenital contractures with pterygia at birth and severe OI-like osteopenia and multiple fractures was reported; and he was shown to have a new homozygous mutation leading to an Arg598His substitution (c.1856G > A) in *PLOD2* (6). This variant point and p. Gly622Cys (c.1864G > T), p. Gly622Val (c.1865G > T), p. Val627Ala (c.1880 T > C), and p. Thr629Ile (c.1886C > T) were all clustered in a stretch of amino acid sequence (1, 6, 8, 41). This amino acid sequence has high homology among three human LH isozymes and LH isozymes of different species (8). In addition, some studies have shown that the residues 610–629 are the LH catalytic domain, and approximately 50% of the identified *PLOD2* variants were located in a narrow region of the domain (42). Therefore, this domain is also considered an important functional domain of LH2 (8).

In the protein solution colloid system, the like charges on the protein surface repel each other and reach a dynamic equilibrium, which plays an important role in maintaining the stability of the colloidal particles. The c.1856G > A variant of this study led to a change from Arg to His at position 619 of the PLOD2 protein. Actually, the difference in physicochemical properties between Arg and His is very small, both are positively charged. The main difference is the pH at which they can lose the positive charge. Arg is the most basic amino acid, which is completely protonated under physiological conditions, that is, it always has a positive charge. However, His is the one whose pKa value of the side chain group is closest to the physiological pH value, and it can become electrically neutral within the physiological range of pH. Therefore, the change of Arg to His can have an effect on protein structure and interactions at different pH. In this study, our multiple RT-qPCR experiments showed that in HEK293T cells transfected with the expression plasmid, the transcription level of the mutated *PLOD2* was significantly increased, while the PLOD2 protein was almost not expressed. We consider that the c.1856G > A variant may affect protein expression at the translation level, while low protein expression may increase mRNA expression through negative feedback regulation.

In the bone tissues of patients with BS, *PLOD2* variant decreased the activity of LH2b, resulting in insufficient hydroxylation of the collagen telopeptide Lys residues, which in turn leads to a decrease in the level of bone collagen pyridinoline cross-linking (1, 12, 37). Hydroxylysyl pyridinoline (HP) and lysyl pyridinoline (LP) are the two main mature cross-linking forms in the bone. HP is mainly distributed in bone and cartilage, with little content in other tissues, whereas LP mainly exists in bone (43). During bone resorption, HP and LP enter directly into the blood circulation and are excreted directly into urine without liver metabolism; therefore, they are sensitive indicators of collagen metabolites in urine (44). Due to their distribution and metabolic characteristics, urinary HP and LP concentrations as biochemical indicators of bone resorption have received increasing attention, and their ratio is a good predictor of bone strength (6, 45). In normal bones, the ratio of HP to LP is approximately 2:1–4:1; however, in patients with BS, HP and LP may be inversely proportional (0.3:1) (7, 46). Some studies have shown that the levels of HP and LP in bone type I collagen in patients with BS were significantly decreased, while the pyridinium levels of ligament type I collagen and cartilage type II collagen were generally normal, indicating that BS may have tissue specificity; only the bone has abnormal cross-linking (27). The loss of normal cross-linking of bone collagen can lead to abnormal bone mineralization and increased bone fragility, resulting in joint contracture or multiple fractures over time (14, 20). The age at onset of joint contracture seems to have great variability (6). Because multiple joint contractures may increase the incidence of fracture and complicate fracture healing, the prognosis of BS patients with severe joint contracture may be worse (4). It should be noted that *PLOD2* variants only cause OI without joint contracture have also been reported (18, 41, 47).

For patients with BS, reducing the incidence of fractures, preventing severe bone deformities, promoting growth, and improving the quality of life are the main treatment objectives. Due to the rarity of BS, there is no obvious breakthrough in its treatment at present, and the main therapy is roughly the same as that of OI. For patients with severe bone structural changes, the corresponding surgical methods can be used to correct the deformity (48). Nowadays, the use of bisphosphates in the treatment of OI or osteoporosis has been widely accepted. Bisphosphate reduces bone resorption by inhibiting osteoclast activity and inducing apoptosis (49). Oral or intravenous bisphosphate has been proven to effectively reduce the risk of fracture, improve bone mineral density, and relieve pain to a certain extent (48). Other non-surgical treatments include vitamin D supplementation to maintain adequate calcium levels, administering growth hormone to promote growth, PTH to promote bone synthesis, and the inhibition of receptor activator of nuclear factor kappa-B ligand to inhibit bone resorption and increase bone mineral density (15, 49). However, the above treatments cannot reverse the severity of BS and the progression of scoliosis, and the curative effect of these treatments on some patients with BS is poor. For example, the proband in this study received bisphosphate regularly to improve bone mineral density, but still had recurrent fractures and progressive scoliosis. The treatment plan based on the patient’s gene diagnosis results may be more conducive to the diagnosis, treatment, and prognosis evaluation of BS, which may be the focus of future research.

## Conclusion

In conclusion, BS is an autosomal recessive hereditary disease characterized by joint contracture and repeated fractures. Gene detection can assist in the early diagnosis of suspected BS. Although the c.1856G > A variant has been found in the past, its function has not been identified and its pathogenicity remains to be confirmed. It is worth noting that this study carried out genetic analysis and functional identification of a family with BS, and c.1856G > A in exon 17 of *PLOD2* was preliminarily confirmed to be a pathogenic variant, which can lead to protein instability. It will provide some data for the early diagnosis of rare disease BS, and provide some ideas for future studies on genotype-based diagnosis and treatment strategies for BS. However, BS is a rare disease, the reported *PLOD2* variant points are few and relatively concentrated, and the function of each domain of the LH2 protein is unclear (38). Therefore, the molecular genetic mechanism of BS which involves *PLOD2* variant remains to be studied, and it is hoped that our research data can provide some reference for it.

## Data Availability Statement

The data presented in this study are deposited to the GenBank under accession number SCV001837654 (https://submit.ncbi.nlm.nih.gov/subs/clinvar_wizard/SUB10342987/overview).

## Ethics Statement

The studies involving human participants were reviewed and approved by the Ethics Committee of Fujian Provincial Hospital. Written informed consent to participate in this study was provided by the participants’ legal guardian/next of kin.

## Author Contributions

F-QT, W-BH, and J-WL conceived and designed the study. J-WL, Y-MG, and X-FL conducted the data analysis. R-LW, D-DR, and Y-NH performed the sample collection and cell experiment. R-LW and D-DR wrote the manuscript. Z-TF and L-SL revised the manuscript and provided some recommendations. All authors read and approved the final manuscript.

## Conflict of Interest

The authors declare that the research was conducted in the absence of any commercial or financial relationships that could be construed as a potential conflict of interest.

## Publisher’s Note

All claims expressed in this article are solely those of the authors and do not necessarily represent those of their affiliated organizations, or those of the publisher, the editors and the reviewers. Any product that may be evaluated in this article, or claim that may be made by its manufacturer, is not guaranteed or endorsed by the publisher.

## Footnotes

1
http://genetics.bwh.harvard.edu/pph2/

2
http://sift.jcvi.org/

3
http://mutationtaster.org/

4
https://alphafold.ebi.ac.uk/entry/O00469

5
http://www.hgmd.org

## Supplementary Material

Supplementary Material 1 The sequence of wild type (WT) plasmid pCDH-CMV-hPLOD2-EF1- copGFP-T2A-Puro.

Supplementary Material 2 The sequence of variant plasmid pCDH-CMV-hPLOD2 mut-EF1-copGFP-T2A-Puro.

Supplementary Material 3 The names of the repository/repositories and accession number(s).

## Abbreviations

BS: bruck syndrome
PTH: parathyroid hormone
25-(OH) D: 25-hydroxyvitamin D
OI: osteogenesis imperfecta
AR-OI: autosomal recessive OI
LH: lysyl hydroxylase
LH2: lysyl hydroxylase 2
RT-Qpcr: Real-time quantitative polymerase chain reaction
PolyPhen-2: polymorphism phenotyping
SIFT: sorting intolerant from Tolerant
NGS: next-generation sequencing
WT: wild type
GFP: green fluorescent protein
PVDF: polyvinylidene fluoride
TBST: tris buffered saline tween
PBS: phosphate buffered saline with 0.05% TWEEN^®^ 20
Lys: lysine
Hyl: hydroxylysine
PPIase: peptidyl-prolyl cis-transisomerase
HP: hydroxylysyl pyridinoline
LP: lysyl pyridinoline.
