# Supplementary material for: Genetic Analysis and Functional Study of a Pedigree With Bruck Syndrome Caused by PLOD2 Variant
Source: Front Pediatr. 2022 May 6;10:878172. doi: 10.3389/fped.2022.878172 (PMC9120662; doi:10.3389/fped.2022.878172)
Supplement: Supplementary Material 1 — The sequence of wild type (WT) plasmid pCDH-CMV-hPLOD2-EF1- copGFP-T2A-Puro. [file Data_Sheet_1.PDF]

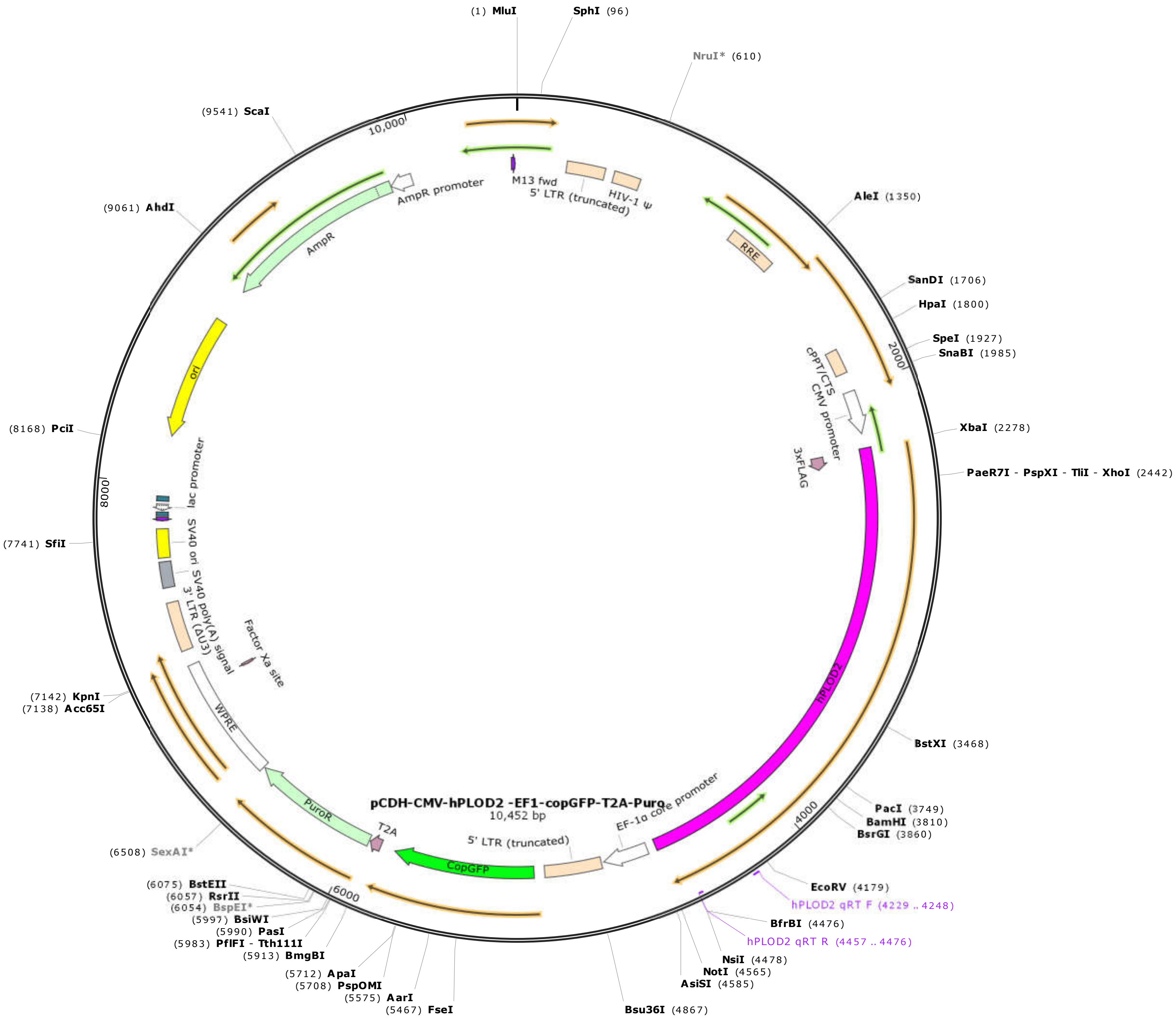
pCDH-CMV-hPLOD2 -EF1-copGFP-T2A-Puro
10,452 bp
(1) MluI
SphI (96)
NruI* (610)
AleI (1350)
SanDI (1706)
HpaI (1800)
SpeI (1927)
SnaBI (1985)
XbaI (2278)
PaeR7I - PspXI - TliI - XhoI (2442)
BstXI (3468)
PacI (3749)
BamHI (3810)
BsrGI (3860)
EcoRV (4179)
hPLOD2 qRT F (4229 .. 4248)
BfrBI (4476)
hPLOD2 qRT R (4457 .. 4476)
NsiI (4478)
NotI (4565)
AsiSI (4585)
Bsu36I (4867)
(5467) FseI
(5575) AarI
(5708) PspOMI
(5712) ApaI
(5913) BmgBI
(5983) PflFI - Tth111I
(5990) PasI
(5997) BsiWI
(6054) BspEI*
(6057) RsrII
(6075) BstEII
(6508) SexAI*
(7138) Acc65I
(7142) KpnI
(7741) SfiI
(8168) PciI
(9061) AhdI
(9541) ScaI
10,000
2000
4000
6000
8000
M13 fwd
5' LTR (truncated)
HIV-1 ψ
RRE
cPPT/CTS
CMV promoter
3xFLAG
hPLOD2
EF-1α core promoter
5' LTR (truncated)
CopGFP
T2A
PuroR
WPRE
Factor Xa site
3' LTR (ΔU3)
SV40 poly(A) signal
SV40 ori
lac promoter
ori
AmpR
AmpR promoter

MluI SphI

5' acgcgtgtagtcttatgcaatactcttgtagtcttgcaacatggtaacgatgagttagcaacatgccttacaaggagagaaaaagcaccgtgcatgccgattggtggaagtaagg 115
3' tgcgcacatcagaatacgttatgagaacatcagaacgttgtaccattgctactcaatcgttgtacggaatgttcctctctttttcgtggcacgtacggctaaccaccttcattcc
T R V V L C N T L V V L Q H G N D E L A T C L T R R E K A P C M P I G G S K
R T T K H L V R T T K C C P L S S N A V H R V L L S F A G H M G I P P L L T

tggtacgatcgtgccttattaggaaggcaacagacgggtctgacatggattggacgaaccactgaattgccgcattgcagagatattgtatttaagtgcctagctcgatacaata 230
accatgctagcacggaataatccttccgttgtctgcccagactgtacctaacctgcttggtgacttaacggcgtaacgtctctataacataaattcacggatcgagctatgttat
V V R S C L I R K A T D G S D M D W T N H
T R D H R I L F A V S P D S M

aacgggtctctctggttagaccagatctgagcctgggagctctctggctaactagggaacccactgcttaagcctcaataaagcttgccttgagtgcttcaagtagtgtgtgccc 345
ttgcccagagagaccaatctggtctagactcggaccctcgagagaccgattgatcccttgggtgacgaattcggagttatttcgaacggaactcacgaagttcatcacacacggg
5' LTR (truncated)

gtctgttgtgtgactctggtaactagagatccctcagacccttttagtcagtgtggaaaatctctagcagtggcgcccgaacagggacctgaaagcgaaagggaaaccagagctc 460
cagacaacacactgagaccattgatctctagggagtctgggaaaatcagtcacaccttttagagatcgtcaccgcgggcttgtccctggactttcgctttccctttggtctcgag
5' LTR (truncated)
HIV-1 Ψ

tctcgacgcaggactcggcttgctgaagcgcgcacggcaagaggcgaggggcggcgactggtgagtacgccaaaaatttttgactagcggaggctagaaggagagagatgggtgcg 575
agagctgcgtcctgagccgaacgacttcgcgcgtgccgttctccgctccccgccgctgaccactcatgcggtttttaaaaactgatcgcctccgatcttcctctctctacccacgc
HIV-1 Ψ

NruI*

agagcgtcagtattaagcgggggagaattagatcgcgatgggaaaaaattcggttaaggccagggggaaagaaaaaatataaattaaaacatatagtatgggcaagcagggagct 690
tctcgcagtcataattcgccccctcttaatctagcgctaccctttttaagccaattccggtccccctttcttttttatatttaattttgtatatcatacccgttcgtccctcga
HIV-1 Ψ

agaacgattcgcagttaatcctggcctgttagaaacatcagaaggctgtagacaaatactgggacagctacaaccatcccttcagacaggatcagaagaacttagatcattatat 805
tcttgctaagcgtcaattaggaccggacaatctttgtagtcttccgacatctgtttatgaccctgtcgatgttggtagggaagtctgtcctagtcttcttgaatctagtaatata

aatacagtagcaaccctctattgtgtgcatcaaaggatagagataaaagacaccaaggaagctttagacaagatagaggaagagcaaaacaaaagtaagaccaccgcacagcaag 920
ttatgtcatcgttgggagataacacacgtagtttcctatctctattttctgtggttccttcgaaatctgttctatctccttctcgttttgttttcattctggtggcgtgtcgttc
V L Y L F L L V F T L G G C L L

cggccactgatcttcagacctggaggaggagatatgagggacaattggagaagtgaattatataaatataaagtagtaaaaattgaaccattaggagtagcacccaccaaggcaa 1035
gccggtgactagaagtctggacctcctcctctatactccctgttaacctcttcacttaatatatttatatttcatcatttttaacttggtaatcctcatcgtgggtggttccgtt
M R D N W R S E L Y K Y K V V K I E P L G V A P T K A
R G S I K L G P P P S I L S L Q L L S N Y L Y L T T F I S G N P T A G V L A F

agagaagagtggtgcagagagaaaaaagagcagtgggaataggagctttgttccttgggttcttgggagcagcaggaagcactatgggcgcagcctcaatgacgctgacggtaca 1150
tctcttctcaccacgtctctcttttttctcgtcacccttatcctcgaaacaaggaacccaagaaccctcgtcgtccttcgtgatacccgcgtcggagttactgcgactgccatgt
RRE
K R R V V Q R E K R A V G I G A L F L G F L G A A G S T M G A A S M T L T V Q
L L T T C L S F L A T P I P A K N R P N K P A A P L V I P A A E I V S V T C

ggccagacaattattgtctggtatagtgcagcagcagaacaatttgctgagggctattgaggcgcaacagcatctgttgcaactcacagtctggggcatcaagcagctccaggca 1265
ccggtctgttaataacagaccatatcacgtcgtcgtcttgttaaacgactcccgataactccgcgttgtcgtagacaacgttgagtgtcagaccccgtagttcgtcgaggtccgt
RRE
A R Q L L S G I V Q Q Q N N L L R A I E A Q Q H L L Q L T V W G I K Q L Q A
A L C N N D P I T C C C F L K S L A I S A C C C R N C S V T Q P M

AleI

agaatcctggctgtggaaagatacctaaaggatcaacagctcctggggatttggggttgctctggaaaactcatttgcaccactgctgtgccttggaatgctagttggagtaata 1380
tcttaggaccgacacctttctatggatttcctagttgtcgaggacccctaaaccccaacgagaccttttgagtaaacgtggtgacgacacggaaccttacgatcaacctcattat
RRE
R I L A V E R Y L K D Q Q L L G I W G C S G K L I C T T A V P W N A S W S N

aatctctggaacagattggaatcacacgacctggatggagtgggacagagaaattaacaattacacaagcttaatacactccttaattgaagaatcgcaaaaccagcaagaaaag 1495
ttagagaccttgtctaaccttagtgtgctggacctacctcaccctgtctctttaattgttaatgtgttcgaattatgtgaggaattaacttcttagcgttttggtcgttcttttc
M E W D R E I N N Y T S L I H S L I E E S Q N Q Q E K
K S L E Q I G I T R P G W S G T E K L T I T Q A

aatgaacaagaattattggaattagataaatgggcaagtttgtggaattggtttaacataacaaattggctgtggtatataaaattattcataatgatagtaggaggcttggtag 1610
ttacttgttcttaataaccttaatctatttacccgttcaaacaccttaaccaaattgtattgtttaaccgacaccatatattttaataagtattactatcatcctccgaaccatc
N E Q E L L E L D K W A S L W N W F N I T N W L W Y I K L F I M I V G G L V

SanDI

gtttaagaatagtttttgctgtactttctatagtgaatagagttaggcagggatattcaccattatcgtttcagacccacctcccaaccccgaggggacccgacaggcccgaagg 1725
caaattcttatcaaaaacgacatgaaagatatcacttatctcaatccgtccctataagtggtaatagcaaagtctgggtggagggttggggctcccctgggctgtccgggcttcc
G L R I V F A V L S I V N R V R Q G Y S P L S F Q T H L P T P R G P D R P E G

HpaI

aatagaagaagaaggtggagagagagacagagacagatccattcgattagtgaacggatctcgacggtatcggttaactttaaaagaaaaggggggattggggggtacagtgca 1840

ttatcttcttcttccacctctctctctgtctctgtctaggtaagctaatcacttgcctagagctgccatagccaattgaaaattttcttttcccccctaaccccccatgtcacgt

cPPT/CTS

I E E E G G E R D R D R S I R L V N G S R R Y R L T F K R K G G I G G Y S A

SpeI

ggggaaagaatagtagacataatagcaacagacatacaaactaaagaattacaaaaacaaattacaaaattcaaaattttatcgatactagtattatgcccagtacatgacctta 1955

ccccttcttatcatctgtattatcgttgtctgtatgtttgatttcttaatgtttttgtttaatgttttaagttttaaaatagctatgatcataatacgggtcatgtactggaat

cPPT/CTS

G E R I V D I I A T D I Q T K E L Q K Q I T K F K I L S I L V L C P V H D L

SnaBI

tgggactttcctacttggcagtacatctacgtattagtcatcgctattaccatggtgatgcggttttggcagtacatcaatgggcgtggatagcggtttgactcacggggatttc 2070

accctgaaaggatgaaccgtcatgtagatgcataatcagtagcgataatggtaccactacgccaaaaccgtcatgtagttacccgcacctatcgccaaactgagtgcccctaaag

CMV promoter

M G L S Y L A V H L R I S H R Y Y H G D A V L A V H Q W A W I A V *

caagtctccaccccattgacgtcaatgggagtttgttttggcaccaaaatcaacgggactttccaaaatgtcgtaacaactccgccccattgacgcaaatgggcggtaggcgtgt 2185

gttcagaggtggggtaactgcagttaccctcaaacaaaaccgtggttttagttgccctgaaaggttttacagcattgttgaggcggggtaactgcgtttacccgccatccgcaca

CMV promoter

* H S N T K A G F D V P S E L I D Y C S R G M S A F P R Y A H

XbaI

acggtgggaggtttatataagcagagctcgtttagtgaaccgtcagatcgcctggagacgccatccacgctgttttgacctccatagaagattctagaatgGACTACAAAGACCA 2300

tgccaccctccaaatatattcgtctcgagcaaatcacttggcagtctagcggacctctgcggtaggtgcgacaaaactggaggtatcttctaagatcttacCTGATGTTTCTGGT

1 5

D Y K D H

CMV promoter

3xFLAG

hPLOD2

M D Y K D H

V T P P K Y L C L E N L S G D S R R S V G D V S N Q G G Y F I R S H V V F V M

TGACGGTGATTATAAAGATCATGACATCGATTACAAGGATGACGACGACAAGgggggatgcacggtgaagcctcagctgctgctcctggcgctcgtcctccacccctggaatccc 2415

ACTGCCACTAATATTTCTAGTACTGTAGCTAATGTTCCTACTGCTGCTGTTCccccctacgtgccacttcggagtcgacgacgaggaccgcgagcaggaggtggggaccttaggg

10 15 20

D G D Y K D H D I D Y K D D D D K G G C T V K P Q L L L L A L V L H P W N P

3xFLAG (in frame with 3xFLAG)

hPLOD2

D G D Y K D H D I D Y K D D D D K G G C T V K P Q L L L L A L V L H P W N P

V T I I F I M

TliI
XhoI
PspXI
PaeR7I

tgtctgggtgcggactcggagaagccctcgagcatccccacagataaattattagtcataactgtagcaacaaaagaaagtgatggattccatcgatttatgcagtcagccaaat 2530

acagacccacgcctgagcctcttcgggagctcgtaggggtgtctatttaataatcagtattgacatcgttgttttctttcactacctaaggtagctaaatacgtcagtcggttta

C L G A D S E K P S S I P T D K L L V I T V A T K E S D G F H R F M Q S A K

(in frame with 3xFLAG)

hPLOD2

C L G A D S E K P S S I P T D K L L V I T V A T K E S D G F H R F M Q S A K

atttcaattatactgtgaaggtccttggtcaaggagaagaatggagaggtggtgatggaattaatagtattggagggggccagaaagtgagattaatgaaagaagtcatggaaca 2645

taaagttaatatgacacttccaggaaccagttcctcttcttacctctccaccactaccttaattatcataacctcccccggtctttcactctaattactttcttcagtaccttgt

Y F N Y T V K V L G Q G E E W R G G D G I N S I G G G Q K V R L M K E V M E H

(in frame with 3xFLAG)

hPLOD2

Y F N Y T V K V L G Q G E E W R G G D G I N S I G G G Q K V R L M K E V M E H

ctatgctgatcaagatgatctggttgtcatgtttactgaatgctttgatgtcatatttgctggtggtccagaagaagttctaaaaaaattccaaaaggcaaaccacaaagtggtc 2760

gatacgactagttctactagaccaacagtacaaatgacttacgaaactacagtataaacgaccaccaggtcttcttcaagatttttttaaggttttccgtttggtgtttcaccag

Y A D Q D D L V V M F T E C F D V I F A G G P E E V L K K F Q K A N H K V V

(in frame with 3xFLAG)

hPLOD2

Y A D Q D D L V V M F T E C F D V I F A G G P E E V L K K F Q K A N H K V V

tttgcagcagatggaattttgtggccagataaaagactagcagacaagtatcctgttgtgcacattgggaaacgctatctgaattcaggaggatttattggctatgctccatatg 2875

aaacgtcgtctaccttaaaacaccggtctattttctgatcgtctgttcataggacaacacgtgtaaccctttgcgatagacttaagtcctcctaaataaccgatacgaggtatac

F A A D G I L W P D K R L A D K Y P V V H I G K R Y L N S G G F I G Y A P Y

(in frame with 3xFLAG)

hPLOD2

F A A D G I L W P D K R L A D K Y P V V H I G K R Y L N S G G F I G Y A P Y

tcaaccgtatagttcaacaatggaatctccaggataatgatgatgatcagctcttttacactaaagtttacattgatccactgaaaagggaagctattaacatcacattggatca 2990
agttggcatatcaagttgttaccttagaggtcctattactactactagtcgagaaaatgtgatttcaaatgtaactaggtgacttttcccttcgataattgtagtgtaacctagt
V N R I V Q Q W N L Q D N D D D Q L F Y T K V Y I D P L K R E A I N I T L D H
(in frame with 3xFLAG)
hPLOD2
V N R I V Q Q W N L Q D N D D D Q L F Y T K V Y I D P L K R E A I N I T L D H

caaatgcaaaattttccagaccttaaatggagctgtagatgaagttgttttaaaatttgaaaatggcaaagccagagctaagaatacattttatgaaacattaccagtggcaatt 3105
gtttacgttttaaaaggtctggaatttacctcgacatctacttcaacaaaattttaaacttttaccgtttcggtctcgattcttatgtaaaatactttgtaatggtcaccgttaa
K C K I F Q T L N G A V D E V V L K F E N G K A R A K N T F Y E T L P V A I
(in frame with 3xFLAG)
hPLOD2
K C K I F Q T L N G A V D E V V L K F E N G K A R A K N T F Y E T L P V A I

aatggaaatggacccaccaagattctcctgaattattttggaaactatgtacccaattcatggacacaggataatggctgcactctttgtgaattcgatacagtcgacttgtctg 3220
ttacctttacctgggtggttctaagaggacttaataaaacctttgatacatgggttaagtacctgtgtcctattaccgacgtgagaaacacttaagctatgtcagctgaacagac
N G N G P T K I L L N Y F G N Y V P N S W T Q D N G C T L C E F D T V D L S
(in frame with 3xFLAG)
hPLOD2
N G N G P T K I L L N Y F G N Y V P N S W T Q D N G C T L C E F D T V D L S

cagtagatgtccatccaaacgtatcaataggtgtttttattgagcaaccaaccccttttctacctcggtttctggacatattgttgacactggattacccaaaagaagcacttaa 3335
gtcatctacaggtaggtttgcatagttatccacaaaaataactcgttggttggggaaaagatggagccaaagacctgtataacaactgtgacctaatgggttttcttcgtgaatt
A V D V H P N V S I G V F I E Q P T P F L P R F L D I L L T L D Y P K E A L K
(in frame with 3xFLAG)
hPLOD2
A V D V H P N V S I G V F I E Q P T P F L P R F L D I L L T L D Y P K E A L K

acttttattcataacaaagaagtttatcatgaaaaggacatcaaggtatttttgataaagctaagcatgaaatcaaaactataaaaatagtaggaccagaagaaaatctaagt 3450
tgaaaataagtattgtttcttcaaatagtacttttcctgtagttccataaaaactatttcgattcgtactttagttttgatatttttatcatcctggtcttcttttagattca
L F I H N K E V Y H E K D I K V F F D K A K H E I K T I K I V G P E E N L S
(in frame with 3xFLAG)
hPLOD2
L F I H N K E V Y H E K D I K V F F D K A K H E I K T I K I V G P E E N L S

BstXI
caagcggaagccagaaacatgggaatggacttttgccgtcaggatgaaaagtgtgattattactttagtgtggatgcagatgttgttttgacaaatccaaggactttaaaaattt 3565
gttcgccttcggtctttgtacccttacctgaaaacggcagtcctacttttcacactaataatgaaatcacacctacgtctacaacaaaactgtttaggttcctgaaatttttaaa
Q A E A R N M G M D F C R Q D E K C D Y Y F S V D A D V V L T N P R T L K I
(in frame with 3xFLAG)
hPLOD2
Q A E A R N M G M D F C R Q D E K C D Y Y F S V D A D V V L T N P R T L K I

tgattgaacaaaacagaaagatcattgctcctcttgtaactcgtcatggaaagctgtggtccaatttctggggagcattgagtcctgatggatactatgcacgatctgaagatta 3680
actaacttgttttgtctttctagtaacgaggagaacattgagcagtacctttcgacaccaggttaaagacccctcgtaactcaggactacctatgatacgtgctagacttctaat
L I E Q N R K I I A P L V T R H G K L W S N F W G A L S P D G Y Y A R S E D Y
(in frame with 3xFLAG)
hPLOD2
L I E Q N R K I I A P L V T R H G K L W S N F W G A L S P D G Y Y A R S E D Y

PacI
tgtggatattgttcaagggaatagagtaggagtatggaatgtcccatatatggctaatgtgtacttaattaaaggaaagacactccgatcagagatgaatgaaaggaactatttt 3795
acacctataacaagttcccttatctcatcctcataccttacagggtatataccgattacacatgaattaatttcctttctgtgaggctagtctctacttactttccttgataaaa
V D I V Q G N R V G V W N V P Y M A N V Y L I K G K T L R S E M N E R N Y F
(in frame with 3xFLAG)
hPLOD2
V D I V Q G N R V G V W N V P Y M A N V Y L I K G K T L R S E M N E R N Y F

BamHI BsrGI
gttcgtgataaactggatcctgatatggctctttgccgaaatgctagagaaatgggtgtatttatgtacatttctaatagacatgaatttggaaggctattatccactgctaatt 3910
caagcactatttgacctaggactataccgagaaacggctttacgatctctttacccacataaatacatgtaaagattatctgtacttaaaccttccgataataggtgacgattaa
V R D K L D P D M A L C R N A R E M G V F M Y I S N R H E F G R L L S T A N
(in frame with 3xFLAG)
hPLOD2
V R D K L D P D M A L C R N A R E M G V F M Y I S N R H E F G R L L S T A N

acaatacttcccattataacaatgacctctggcagatttttgaaaatcctgtggactggaaggaaaagtatataaaccgtgattattcaaagatttttcactgaaaatatagttga 4025
tgttatgaagggtaatattgttactggagaccgtctaaaaactttaggacacctgaccttccttttcatatatttggcactaataagtttctaaaagtgacttttatatcaact
Y N T S H Y N N D L W Q I F E N P V D W K E K Y I N R D Y S K I F T E N I V E
(in frame with 3xFLAG)
hPLOD2
Y N T S H Y N N D L W Q I F E N P V D W K E K Y I N R D Y S K I F T E N I V E
× L N E S F I Y N F

```
acagccctgtccagatgtcttttggttccccatattttctgaaaaagcctgtgatgaattggtagaagaaatggaacattacggcaaatggtctgggggaaaacatcatgatagc   4140
tgtcgggacaggtctacagaaaaccaaggggtataaaagactttttcggacactacttaaccatcttctttaccttgtaatgccgtttaccagacccccttttgtagtactatcg
 Q  P  C  P  D  V  F  W  F  P  I  F  S  E  K  A  C  D  E  L  V  E  E  M  E  H  Y  G  K  W  S  G  G  K  H  H  D  S
(in frame with 3xFLAG)
hPLOD2
 Q  P  C  P  D  V  F  W  F  P  I  F  S  E  K  A  C  D  E  L  V  E  E  M  E  H  Y  G  K  W  S  G  G  K  H  H  D  S  →
← L  G  T  W  I  D  K  P  E  G  Y  K  R  F  F  G  T  I  F  Q  Y  F  F  H  F  M  V  A  F  P  R  P  S  F  M  M  I  A

                                 EcoRV                                                          hPLOD2 qRT F
                                                                                                gggagttcattgcaccagtt
cgtatatctggtggttatgaaaatgtcccaactgatgatatccacatgaagcaagttgatctggagaatgtatggcttcattttatccgggagttcattgcaccagttacactga   4255
gcatatagaccaccaatacttttacagggttgactactataggtgtacttcgttcaactagacctcttacataccgaagtaaaataggccctcaagtaacgtggtcaatgtgact
 R  I  S  G  G  Y  E  N  V  P  T  D  D  I  H  M  K  Q  V  D  L  E  N  V  W  L  H  F  I  R  E  F  I  A  P  V  T  L
(in frame with 3xFLAG)
hPLOD2
 R  I  S  G  G  Y  E  N  V  P  T  D  D  I  H  M  K  Q  V  D  L  E  N  V  W  L  H  F  I  R  E  F  I  A  P  V  T  L  →
← T  Y  R  T  T  I  F  I  D  W  S  I  I  D  V  H  L  L  N  I  Q  L  I  Y  P  K  M

aggtctttgcaggctattatacgaagggatttgcactactgaattttgtagtaaaatactcccctgaacgacagcgttctcttcgtcctcatcatgatgcttctacatttaccat   4370
tccagaaacgtccgataatatgcttccctaaacgtgatgacttaaaacatcattttatgaggggacttgctgtcgcaagagaagcaggagtagtactacgaagatgtaaatggta
K  V  F  A  G  Y  Y  T  K  G  F  A  L  L  N  F  V  V  K  Y  S  P  E  R  Q  R  S  L  R  P  H  H  D  A  S  T  F  T  I
(in frame with 3xFLAG)
hPLOD2
K  V  F  A  G  Y  Y  T  K  G  F  A  L  L  N  F  V  V  K  Y  S  P  E  R  Q  R  S  L  R  P  H  H  D  A  S  T  F  T  I  →

                                                                                                         BfrBI  NsiI
aaacattgcacttaataacgtgggagaagactttcagggaggtggttgcaaatttctaaggtacaattgctctattgagtcaccacgaaaaggctggagcttcatgcatcctggg   4485
tttgtaacgtgaattattgcaccctcttctgaaagtccctccaccaacgtttaaagattccatgttaacgagataactcagtggtgcttttccgacctcgaagtacgtaggaccc
 N  I  A  L  N  N  V  G  E  D  F  Q  G  G  G  C  K  F  L  R  Y  N  C  S  I  E  S  P  R  K  G  W  S  F  M  H  P  G
(in frame with 3xFLAG)
hPLOD2
 N  I  A  L  N  N  V  G  E  D  F  Q  G  G  G  C  K  F  L  R  Y  N  C  S  I  E  S  P  R  K  G  W  S  F  M  H  P  G  →
                                                                                         cttttccgacctcgaagtac
                                                                                         hPLOD2 qRT R

                                                                                NotI                  AsiSI
agactcacacatttgcatgaaggacttcctgttaaaaatggaacaagatacattgcagtgtcatttatagatccctaagcggccgcgaaggatctgcgatcgctccggtgcccgt   4600
tctgagtgtgtaaacgtacttcctgaaggacaatttttaccttgttctatgtaacgtcacagtaaatatctagggattcgccggcgcttcctagacgctagcgaggccacgggca
 R  L  T  H  L  H  E  G  L  P  V  K  N  G  T  R  Y  I  A  V  S  F  I  D  P  *
(in frame with 3xFLAG)
hPLOD2
 R  L  T  H  L  H  E  G  L  P  V  K  N  G  T  R  Y  I  A  V  S  F  I  D  P  *                                         →

cagtgggcagagcgcacatcgcccacagtccccgagaagttggggggaggggtcggcaattgaacgggtgcctagagaaggtggcgcggggtaaactgggaaagtgatgtcgtgt   4715
gtcacccgtctcgcgtgtagcgggtgtcaggggctcttcaaccccccctccccagccgttaacttgcccacggatctcttccaccgcgccccatttgaccctttcactacagcaca
EF-1α core promoter

actggctccgcctttttcccgagggtgggggagaaccgtatataagtgcagtagtcgccgtgaacgttctttttcgcaacgggtttgccgccagaacacagctgaagcttcgagg   4830
tgaccgaggcggaaaaagggctcccaccccctcttggcatatattcacgtcatcagcggcacttgcaagaaaaagcgttgcccaaacggcggtcttgtgtcgacttcgaagctcc
EF-1α core promoter
5' LTR (truncated)

                                   Bsu36I
ggctcgcatctctccttcacgcgcccgccgccctacctgaggccgccatccacgccggttgagtcgcgttctgccgcctcccgcctgtggtgcctcctgaactgcgtccgccgtc   4945
ccgagcgtagagaggaagtgcgcgggcggcgggatggactccggcggtaggtgcggccaactcagcgcaagacggcggagggcggacaccacggaggacttgacgcaggcggcag
5' LTR (truncated)

taggtaagtttaaagctcaggtcgagaccgggcctttgtccggcgctcccttggagcctacctagactcagccggctctccacgctttgcctgaccctgcttgctcaactctacg   5060
atccattcaaatttcgagtccagctctggcccggaaacaggccgcgagggaacctcggatggatctgagtcggccgagaggtgcgaaacggactgggacgaacgagttgagatgc
5' LTR (truncated)

tctttgtttcgttttctgttctgcgccgttacagatccaagctgtgaccggcgcctacgctagatggagagcgacgagagcggcctgcccgccatggagatcgagtgccgcatca   5175
agaaacaaagcaaaagacaagacgcggcaatgtctaggttcgacactggccgcggatgcgatctacctctcgctgctctcgccggacgggcggtacctctagctcacggcgtagt
                                                                                            5        10
                                                                                P  A  M  E  I  E  C  R  I
5' LTR (truncated)                                                              CopGFP
                                                         M  E  S  D  E  S  G  L  P  A  M  E  I  E  C  R  I  →

ccggcaccctgaacggcgtggagttcgagctggtgggcggcggagagggcacccccaagcagggccgcatgaccaacaagatgaagagcaccaaaggcgccctgaccttcagccc   5290
ggccgtgggacttgccgcacctcaagctcgaccacccgccgcctctcccgtggggttcgtcccggcgtactggttgttctacttctcgtggtttccgcgggactggaagtcggg
      15        20        25        30        35        40        45
T  G  T  L  N  G  V  E  F  E  L  V  G  G  G  E  G  T  P  K  Q  G  R  M  T  N  K  M  K  S  T  K  G  A  L  T  F  S  P
CopGFP
T  G  T  L  N  G  V  E  F  E  L  V  G  G  G  E  G  T  P  K  Q  G  R  M  T  N  K  M  K  S  T  K  G  A  L  T  F  S  P  →
```

```
ctacctgctgagccacgtgatgggctacggcttctaccacttcggcacctaccccagcggctacgagaaccccttcctgcacgccatcaacaacggcggctacaccaacacccgc 5405
gatggacgactcggtgcactacccgatgccgaagatggtgaagccgtggatggggtcgccgatgctcttggggaaggacgtgcggtagttgttgccgccgatgtggttgtgggcg
50 55 60 65 70 75 80 85
Y L L S H V M G Y G F Y H F G T Y P S G Y E N P F L H A I N N G G Y T N T R
CopGFP
Y L L S H V M G Y G F Y H F G T Y P S G Y E N P F L H A I N N G G Y T N T R
```

```
FseI
atcgagaagtacgaggacggcggcgtgctgcacgtgagcttcagctaccgctacgaggccggccgcgtgatcggcgacttcaaggtggtgggcaccggcttccccgaggacagcg 5520
tagctcttcatgctcctgccgccgcacgacgtgcactcgaagtcgatggcgatgctccggccggcgcactagccgctgaagttccacccgtggccgaaggggctcctgtcgc
90 95 100 105 110 115 120 125
I E K Y E D G G V L H V S F S Y R Y E A G R V I G D F K V V G T G F P E D S
CopGFP
I E K Y E D G G V L H V S F S Y R Y E A G R V I G D F K V V G T G F P E D S
```

```
AarI
tgatcttcaccgacaagatcatccgcagcaacgccaccgtggagcacctgcaccccatgggcgataacgtgctggtgggcagcttcgcccgcaccttcagcctgcgcgacggcgg 5635
actagaagtggctgttctagtaggcgtcgttgcggtggcacctcgtggacgtggggtacccgctattgcacgaccacccgtcgaagcgggcgtggaagtcggacgcgctgccgcc
130 135 140 145 150 155 160
V I F T D K I I R S N A T V E H L H P M G D N V L V G S F A R T F S L R D G G
CopGFP
V I F T D K I I R S N A T V E H L H P M G D N V L V G S F A R T F S L R D G G
```

```
PspOMI ApaI
ctactacagcttcgtggtggacagccacatgcacttcaagagcgccatccaccccagcatcctgcagaacgggggccccatgttcgccttccgccgcgtggaggagctgcacagc 5750
gatgatgtcgaagcaccacctgtcggtgtacgtgaagttctcgcggtaggtggggtcgtaggacgtcttgcccccggggtacaagcggaaggcggcgcacctcctcgacgtgtcg
165 170 175 180 185 190 195 200
Y Y S F V V D S H M H F K S A I H P S I L Q N G G P M F A F R R V E E L H S
CopGFP
Y Y S F V V D S H M H F K S A I H P S I L Q N G G P M F A F R R V E E L H S
```

```
aacaccgagctgggcatcgtggagtaccagcacgccttcaagacccccatcgccttcgccagatcccgcgctcagtcgtccaattctgccgtggacggcaccgccggacccggct 5865
ttgtggctcgacccgtagcacctcatggtcgtgcggaagttctgggggtagcggaagcggtctagggcgcgagtcagcaggttaagacggcacctgccgtggcggcctgggccga
205 210 215 220
N T E L G I V E Y Q H A F K T P I A F A R S R A Q S S N S A V D G T A G P G
CopGFP (in frame with CopGFP)
N T E L G I V E Y Q H A F K T P I A F A R S R A Q S S N S A V D G T A G P G
```

```
BmgBI
ccaccggatctcgctgagggcagaggaagtcttctaacatgcggtgacgtggaggagaatcccggcccttccgggatgaccgagtacaagcccacggtgcgcctcgccacccgcg 5980
ggtggcctagagcgactcccgtctccttcagaagattgtacgccactgcacctcctcttagggccgggaaggccctactggctcatgttcgggtgccacgcggagcggtgggcgc
S T G S R ■
1 5 10
M T E Y K P T V R L A T R
PuroR
140 145 150 155
E G R G S L L T C G D V E E N P G P S G
T2A
M T E Y K P T V R L A T R
S T G S R ■
```

```
PflFI
Tth111I PasI BsiWI BspEI* RsrII BstEII
acgacgtccccagggccgtacgcaccctcgccgccgcgttcgccgactaccccgccacgcgccacaccgtcgatccggaccgccacatcgagcgggtcaccgagctgcaagaact 6095
tgctgcaggggtcccggcatgcgtgggagcggcggcgcaagcggctgatggggcggtgcgcggtgtggcagctaggcctggcggtgtagctcgcccagtggctcgacgttcttga
15 20 25 30 35 40 45 50
D D V P R A V R T L A A A F A D Y P A T R H T V D P D R H I E R V T E L Q E L
PuroR
D D V P R A V R T L A A A F A D Y P A T R H T V D P D R H I E R V T E L Q E L
```

```
cttcctcacgcgcgtcgggctcgacatcggcaaggtgtgggtcgcggacgacggcgccgcggtggcggtctggaccacgccggagagcgtcgaagcgggggcggtgttcgccgag 6210
gaaggagtgcgcgcagcccgagctgtagccgttccacacccagcgcctgctgccgcggcgccaccgccagacctggtgcggcctctcgcagcttcgcccccgccacaagcggctc
55 60 65 70 75 80 85 90
F L T R V G L D I G K V W V A D D G A A V A V W T T P E S V E A G A V F A E
PuroR
F L T R V G L D I G K V W V A D D G A A V A V W T T P E S V E A G A V F A E
```

```
atcggcccgcgcatggccgagttgagcggttcccggctggccgcgcagcaacagatggaaggcctcctggcgccgcaccggcccaaggagcccgcgtggttcctggccaccgtcg 6325
tagccgggcgcgtaccggctcaactcgccaagggccgaccggcgcgtcgttgtctaccttccggaggaccgcggcgtggccgggttcctcgggcgcaccaaggaccggtggcagc
95 100 105 110 115 120 125
I G P R M A E L S G S R L A A Q Q Q M E G L L A P H R P K E P A W F L A T V
PuroR
I G P R M A E L S G S R L A A Q Q Q M E G L L A P H R P K E P A W F L A T V
```

```
gcgtctcgcccgaccaccagggcaagggtctgggcagcgccgtcgtgctccccggagtggaggcggccgagcgcgccggggtgcccgccttcctggagacctccgcgccccgcaa 6440
cgcagagcgggctggtggtcccgttcccagacccgtcgcggcagcacgaggggcctcacctccgccggctcgcgcggccccacgggcggaaggacctctggaggcgcggggcgtt
130 135 140 145 150 155 160 165
G V S P D H Q G K G L G S A V V L P G V E A A E R A G V P A F L E T S A P R N
PuroR
G V S P D H Q G K G L G S A V V L P G V E A A E R A G V P A F L E T S A P R N
```

SexAI*

cctccccttctacgagcggctcggcttcaccgtcaccgccgacgtcgaggtgcccgaaggaccgcgcacctggtgcatgacccgcaagcccggtgcctgagtcgacaatcaacct 6555
ggaggggaagatgctcgccgagccgaagtggcagtggcggctgcagctccacgggcttcctggcgcgtggaccacgtactgggcgttcgggccacggactcagctgttagttgga

170 175 180 185 190 195 200
L P F Y E R L G F T V T A D V E V P E G P R T W C M T R K P G A *
PuroR
WPRE
L P F Y E R L G F T V T A D V E V P E G P R T W C M T R K P G A *

ctggattacaaaatttgtgaaagattgactggtattcttaactatgttgctccttttacgctatgtggatacgctgctttaatgcctttgtatcatgctattgcttcccgtatgg 6670
gacctaatgttttaaacactttctaactgaccataagaattgatacaacgaggaaaatgcgatacacctatgcgacgaaattacggaaacatagtacgataacgaagggcatacc
WPRE
M P L Y H A I A S R M
M L L L P V W

ctttcattttctcctccttgtataaatcctggttgctgtctctttatgaggagttgtggcccgttgtcaggcaacgtggcgtggtgtgcactgtgtttgctgacgcaacccccac 6785
gaaagtaaaagaggaggaacatatttaggaccaacgacagagaaatactcctcaacaccgggcaacagtccgttgcaccgcaccacacgtgacacaaacgactgcgttgggggtg
WPRE
A F I F S S L Y K S W L L S L Y E E L W P V V R Q R G V V C T V F A D A T P T
L S F S P P C I N P G C C L F M R S C G P L S G N V A W C A L C L L T Q P P

tggttggggcattgccaccacctgtcagctcctttccgggactttcgctttccccctccctattgccacggcggaactcatcgccgcctgccttgcccgctgctggacaggggct 6900
accaaccccgtaacggtggtggacagtcgaggaaaggccctgaaagcgaaagggggagggataacggtgccgccttgagtagcggcggacggaacgggcgacgacctgtccccga
WPRE
G W G I A T T C Q L L S G T F A F P L P I A T A E L I A A C L A R C W T G A
L V G A L P P P V S S F P G L S L S P S L L P R R N S S P P A L P A A G Q G L

cggctgttgggcactgacaattccgtggtgttgtcggggaaatcatcgtcctttccttggctgctcgcctgtgttgccacctggattctgcgcgggacgtccttctgctacgtcc 7015
gccgacaacccgtgactgttaaggcaccacaacagcccctttagtagcaggaaaggaaccgacgagcggacacaacggtggacctaagacgcgccctgcaggaagacgatgcagg
* R G K R P Q E G T N G G P N Q A P R G E A V D
(in frame with Factor Xa site)
WPRE
R L L G T D N S V V L S G K S S S F P W L L A C V A T W I L R G T S F C Y V
G C W A L T I P W C C R G N H R P F L G C S P V L P P G F C A G R P S A T S

cttcggccctcaatccagcggaccttccttcccgcggcctgctgccggctctgcggcctcttccgcgtcttcgccttcgccctcagacgagtcggatctccctttgggccgcctc 7130
gaagccgggagttaggtcgcctggaaggaagggcgccggacgacggccgagacgccggagaaggcgcagaagcggaagcgggagtctgctcagcctagagggaaacccggcggag
1
R R G E I
Factor Xa site
WPRE
P S A L N P A D L P S R G L L P A L R P L P R L R L R P Q T S R I S L W A A S
L R P S I Q R T F L P A A C C R L C G L F R V F A F A L R R V G S P F G P P

Acc65I KpnI

cccgcctggtacctttaagaccaatgacttacaaggcagctgtagatcttagccactttttaaaagaaaaggggggactggaagggctaattcactcccaacgaaaataagatct 7245
gggcggaccatggaaattctggttactgaatgttccgtcgacatctagaatcggtgaaaaattttcttttcccccctgaccttcccgattaagtgagggttgcttttattctaga
WPRE
3' LTR (ΔU3)
P P G T F K T N D L Q G S C R S *
P R L V P L R P M T Y K A A V D L S H F L K E K G G L E G L I H S Q R K *

gcttttttgcttgtactgggtctctctggttagaccagatctgagcctgggagctctctggctaactagggaacccactgcttaagcctcaataaagcttgccttgagtgcttcaa 7360
cgaaaaaacgaacatgacccagagagaccaatctggtctagactcggaccctcgagagaccgattgatcccttgggtgacgaattcggagttatttcgaacggaactcacgaagtt
3' LTR (ΔU3)

gtagtgtgtgcccgtctgttgtgtgactctggtaactagagatccctcagacccttttagtcagtgtggaaaatctctagcagtagtagttcatgtcatcttattattcagtatt 7475
catcacacacgggcagacaacacactgagaccattgatctctagggagtctgggaaaatcagtcacaccttttagagatcgtcatcatcaagtacagtagaataataagtcataa
3' LTR (ΔU3)

tataacttgcaaagaaatgaatatcagagagtgagaggaacttgtttattgcagcttataatggttacaaataaagcaatagcatcacaaatttcacaaataaagcatttttttc 7590
atattgaacgtttctttacttatagtctctcactctccttgaacaaataacgtcgaatattaccaatgtttatttcgttatcgtagtgtttaaagtgtttatttcgtaaaaaaag
SV40 poly(A) signal

actgcattctagttgtggtttgtccaaactcatcaatgtatcttatcatgtctggctctagctatcccgcccctaactccgcccagttccgcccattctccgccccatggctgac 7705
tgacgtaagatcaacaccaaacaggtttgagtagttacatagaatagtacagaccgagatcgatagggcggggattgaggcgggtcaaggcgggtaagaggcggggtaccgactg
SV40 poly(A) signal
SV40 ori

SfiI

taatttttttatttatgcagaggccgaggccgcctcggcctctgagctattccagaagtagtgaggaggcttttttggaggcctagacttttgcagagacggcccaaattcgta 7820
attaaaaaaataaatacgtctccggctccggcggagccggagactcgataaggtcttcatcactcctccgaaaaaacctccggatctgaaaacgtctctgccgggtttaagcat
SV40 ori

atcatggtcatagctgtttcctgtgtgaaattgttatccgctcacaattccacacaacatacgagccggaagcataaagtgtaaagcctggggtgcctaatgagtgagctaactc 7935
tagtaccagtatcgacaaaggacacactttaacaataggcgagtgttaaggtgtgttgtatgctcggccttcgtatttcacatttcggaccccacggattactcactcgattgag
M13 rev
lac operator
-10 -35
lac promoter
CAP binding site

acattaattgcgttgcgctcactgcccgctttccagtcgggaaacctgtcgtgccagctgcattaatgaatcggccaacgcgcggggagaggcggtttgcgtattgggcgctctt 8050
tgtaattaacgcaacgcgagtgacgggcgaaaggtcagccctttggacagcacggtcgacgtaattacttagccggttgcgcgcccctctccgccaaacgcataacccgcgagaa

CAP binding site

ccgcttcctcgctcactgactcgctgcgctcggtcgttcggctgcggcgagcggtatcagctcactcaaaggcggtaatacggttatccacagaatcaggggataacgcaggaaa 8165
ggcgaaggagcgagtgactgagcgacgcgagccagcaagccgacgccgctcgccatagtcgagtgagtttccgccattatgccaataggtgtcttagtcccctattgcgtccttt

PciI

gaacatgtgagcaaaaggccagcaaaaggccaggaaccgtaaaaaggccgcgttgctggcgtttttccataggctccgcccccctgacgagcatcacaaaaatcgacgctcaagt 8280
cttgtacactcgttttccggtcgttttccggtccttggcatttttccggcgcaacgaccgcaaaaaggtatccgaggcggggggactgctcgtagtgtttttagctgcgagttca

ori

cagaggtggcgaaacccgacaggactataaagataccaggcgtttccccctggaagctccctcgtgcgctctcctgttccgaccctgccgcttaccggatacctgtccgcctttc 8395
gtctccaccgctttgggctgtcctgatatttctatggtccgcaaagggggaccttcgagggagcacgcgagaggacaaggctgggacggcgaatggcctatggacaggcggaaag

ori

tcccttcgggaagcgtggcgctttctcatagctcacgctgtaggtatctcagttcggtgtaggtcgttcgctccaagctgggctgtgtgcacgaaccccccgttcagcccgaccg 8510
agggaagcccttcgcaccgcgaaagagtatcgagtgcgacatccatagagtcaagccacatccagcaagcgaggttcgacccgacacacgtgcttggggggcaagtcgggctggc

ori

ctgcgccttatccggtaactatcgtcttgagtccaacccggtaagacacgacttatcgccactggcagcagccactggtaacaggattagcagagcgaggtatgtaggcggtgct 8625
gacgcggaataggccattgatagcagaactcaggttgggccattctgtgctgaatagcggtgaccgtcgtcggtgaccattgtcctaatcgtctcgctccatacatccgccacga

ori

acagagttcttgaagtggtggcctaactacggctacactagaaggacagtatttggtatctgcgctctgctgaagccagttaccttcggaaaaagagttggtagctcttgatccg 8740
tgtctcaagaacttcaccaccggattgatgccgatgtgatcttcctgtcataaaccatagacgcgagacgacttcggtcaatggaagcctttttctcaaccatcgagaactaggc

ori

gcaaacaaaccaccgctggtagcggtggtttttttgtttgcaagcagcagattacgcgcagaaaaaaaggatctcaagaagatcctttgatcttttctacggggtctgacgctca 8855
cgtttgtttggtggcgaccatcgccaccaaaaaaacaaacgttcgtcgtctaatgcgcgtctttttttcctagagttcttctaggaaactagaaaagatgccccagactgcgagt

ori

gtggaacgaaaactcacgttaagggattttggtcatgagattatcaaaaaggatcttcacctagatccttttaaattaaaaatgaagttttaaatcaatctaaagtatatatgag 8970
caccttgcttttgagtgcaattccctaaaaccagtactctaatagtttttcctagaagtggatctaggaaaatttaatttttacttcaaaatttagttagatttcatatatactc

AhdI

taaacttggtctgacagttaccaatgcttaatcagtgaggcacctatctcagcgatctgtctatttcgttcatccatagttgcctgactccccgtcgtgtagataactacgatac 9085
atttgaaccagactgtcaatggttacgaattagtcactccgtggatagagtcgctagacagataaagcaagtaggtatcaacggactgaggggcagcacatctattgatgctatg

285 280 275 270 265 260 255
* W H K I L S A G I E A I Q R N R E D M T A Q S G T T Y I V V I R

AmpR

* W H K I L S A G I E A I Q R N R E D M T A Q S G T T Y I V V I R

gggagggcttaccatctggccccagtgctgcaatgataccgcgagacccacgctcaccggctccagatttatcagcaataaaccagccagccggaagggccgagcgcagaagtgg 9200
ccctcccgaatggtagaccggggtcacgacgttactatggcgctctgggtgcgagtggccgaggtctaaatagtcgttatttggtcggtcggccttcccggctcgcgtcttcacc

250 245 240 235 230 225 220
S P K G D P G L A A I I G R S G R E G A G S K D A I F W G A P L A S R L L P

AmpR

M I P R D P R S P A P D L S A I N Q P A G R A E R R S G
S P K G D P G L A A I I G R S G R E G A G S K D A I F W G A P L A S R L L P

tcctgcaactttatccgcctccatccagtctattaattgttgccgggaagctagagtaagtagttcgccagttaatagtttgcgcaacgttgttgccattgctacaggcatcgtg 9315
aggacgttgaaataggcggaggtaggtcagataattaacaacggcccttcgatctcattcatcaagcggtcaattatcaaacgcgttgcaacaacggtaacgatgtccgtagcac

215 210 205 200 195 190 185 180
G A V K D A E M W D I L Q Q R S A L T L L E G T L L K R L T T A M A V P M T

AmpR

P A T L S A S I Q S I N C C R E A R V S S S P V N S L R N V V A I A T G I V
G A V K D A E M W D I L Q Q R S A L T L L E G T L L K R L T T A M A V P M T

gtgtcacgctcgtcgtttggtatggcttcattcagctccggttcccaacgatcaaggcgagttacatgatcccccatgttgtgcaaaaaagcggttagctccttcggtcctccga 9430
cacagtgcgagcagcaaaccataccgaagtaagtcgaggccaagggttgctagttccgctcaatgtactagggggtacaacacgttttttcgccaatcgaggaagccaggaggct

175 170 165 160 155 150 145 140
T D R E D N P I A E N L E P E W R D L R T V H D G M N H L F A T L E K P G G I

AmpR

V S R S S F G M A S F S S G S Q R S R R V T *
T D R E D N P I A E N L E P E W R D L R T V H D G M N H L F A T L E K P G G I

ScaI

tcgttgtcagaagtaagttggccgcagtgttatcactcatggttatggcagcactgcataattctcttactgtcatgccatccgtaagatgcttttctgtgactggtgagtactc 9545
agcaacagtcttcattcaaccggcgtcacaatagtgagtaccaataccgtcgtgacgtattaagagaatgacagtacggtaggcattctacgaaaagacactgaccactcatgag

135 130 125 120 115 110 105
T T L L L N A A T N D S M T I A A S C L E R V T M G D T L H K E T V P S Y E
AmpR

T T L L L N A A T N D S M T I A A S C L E R V T M G D T L H K E T V P S Y E

aaccaagtcattctgagaatagtgtatgcggcgaccgagttgctcttgcccggcgtcaatacgggataataccgcgccacatagcagaactttaaaagtgctcatcattggaaaa 9660
ttggttcagtaagactcttatcacatacgccgctggctcaacgagaacgggccgcagttatgccctattatggcgcggtgtatcgtcttgaaattttcacgagtagtaacctttt

100 95 90 85 80 75 70 65
V L D N Q S Y H I R R G L Q E Q G A D I R S L V A G C L L V K F T S M M P F
AmpR

V L D N Q S Y H I R R G L Q E Q G A D I R S L V A G C L L V K F T S M M P F

cgttcttcggggcgaaaactctcaaggatcttaccgctgttgagatccagttcgatgtaacccactcgtgcacccaactgatcttcagcatcttttactttcaccagcgtttctg 9775
gcaagaagccccgcttttgagagttcctagaatggcgacaactctaggtcaagctacattgggtgagcacgtgggttgactagaagtcgtagaaaatgaaagtggtcgcaaagac

60 55 50 45 40 35 30 25
R E E P R F S E L I K G S N L D L E I Y G V R A G L Q D E A D K V K V L T E P
AmpR

R E E P R F S E L I K G S N L D L E I Y G V R A G L Q D E A D K V K V L T E P

ggtgagcaaaaacaggaaggcaaaatgccgcaaaaaagggaataagggcgacacggaaatgttgaatactcatactcttcctttttcaatattattgaagcatttatcagggtta 9890
ccactcgtttttgtccttccgttttacggcgttttttcccttattcccgctgtgcctttacaacttatgagtatgagaaggaaaaagttataataacttcgtaaatagtcccaat

20 15 10 5 1
H A F V P L C F A A F F P I L A V R F H Q I S M
signal sequence
AmpR
AmpR promoter

H A F V P L C F A A F F P I L A V R F H Q I S M

ttgtctcatgagcggatacatatttgaatgtatttagaaaaataaacaaataggggttccgcgcacatttccccgaaaagtgccacctgacgtctaagaaaccattattatcatg 10,005
aacagagtactcgcctatgtataaacttacataaatctttttatttgtttatccccaaggcgcgtgtaaaggggcttttcacggtggactgcagattctttggtaataatagtac

AmpR promoter

acattaacctataaaaataggcgtatcacgaggccctttcgtctcgcgcgtttcggtgatgacggtgaaaacctctgacacatgcagctcccggagacggtcacagcttgtctgt 10,120
tgtaattggatatttttatccgcatagtgctccgggaaagcagagcgcgcaaagccactactgccacttttggagactgtgtacgtcgagggcctctgccagtgtcgaacagaca

aagcggatgccgggagcagacaagcccgtcagggcgcgtcagcgggtgttggcgggtgtcggggctggcttaactatgcggcatcagagcagattgtactgagagtgcaccatat 10,235
ttcgcctacggccctcgtctgttcgggcagtcccgcgcagtcgcccacaaccgcccacagccccgaccgaattgatacgccgtagtctcgtctaacatgactctcacgtggtata

M
* A A D S C I T S L T C W I

gcggtgtgaaataccgcacagatgcgtaaggagaaaataccgcatcaggcgccattcgccattcaggctgcgcaactgttgggaagggcgatcggtgcgggcctcttcgctatta 10,350
cgccacactttatggcgtgtctacgcattcctcttttatggcgtagtccgcggtaagcggtaagtccgacgcgttgacaacccttcccgctagccacgcccggagaagcgataat

R C E I P H R C V R R K Y R I R R H S P F R L R N C W E G R S V R A S S L L
R H S I G C L H T L L F Y R M L R W E G N L S R L Q Q S P R D T R A E E S N

cgccagctggcgaaagggggatgtgctgcaaggcgattaagttgggtaacgccagggttttcccagtcacgacgttgtaaaacgacggccagtgccaagctg 3' ••• 10,452
gcggtcgaccgctttcccccctacacgacgttccgctaattcaacccattgcggtcccaaaagggtcagtgctgcaacattttgctgccggtcacggttcgac 5'

M13 fwd

R Q L A K G G C A A R R L S W V T P G F S Q S R R C K T T A S A K L
R W S A F P P H A A L R N L Q T V G P N E W D R R Q L V V A L A L S
