# Supplementary material for: Genetic Analysis and Functional Study of a Pedigree With Bruck Syndrome Caused by PLOD2 Variant
Source: Front Pediatr. 2022 May 6;10:878172. doi: 10.3389/fped.2022.878172 (PMC9120662; doi:10.3389/fped.2022.878172)
Supplement: Supplementary Material 2 — The sequence of variant plasmid pCDH-CMV-hPLOD2 mut-EF1-copGFP-T2A-Puro. [file Data_Sheet_2.PDF]

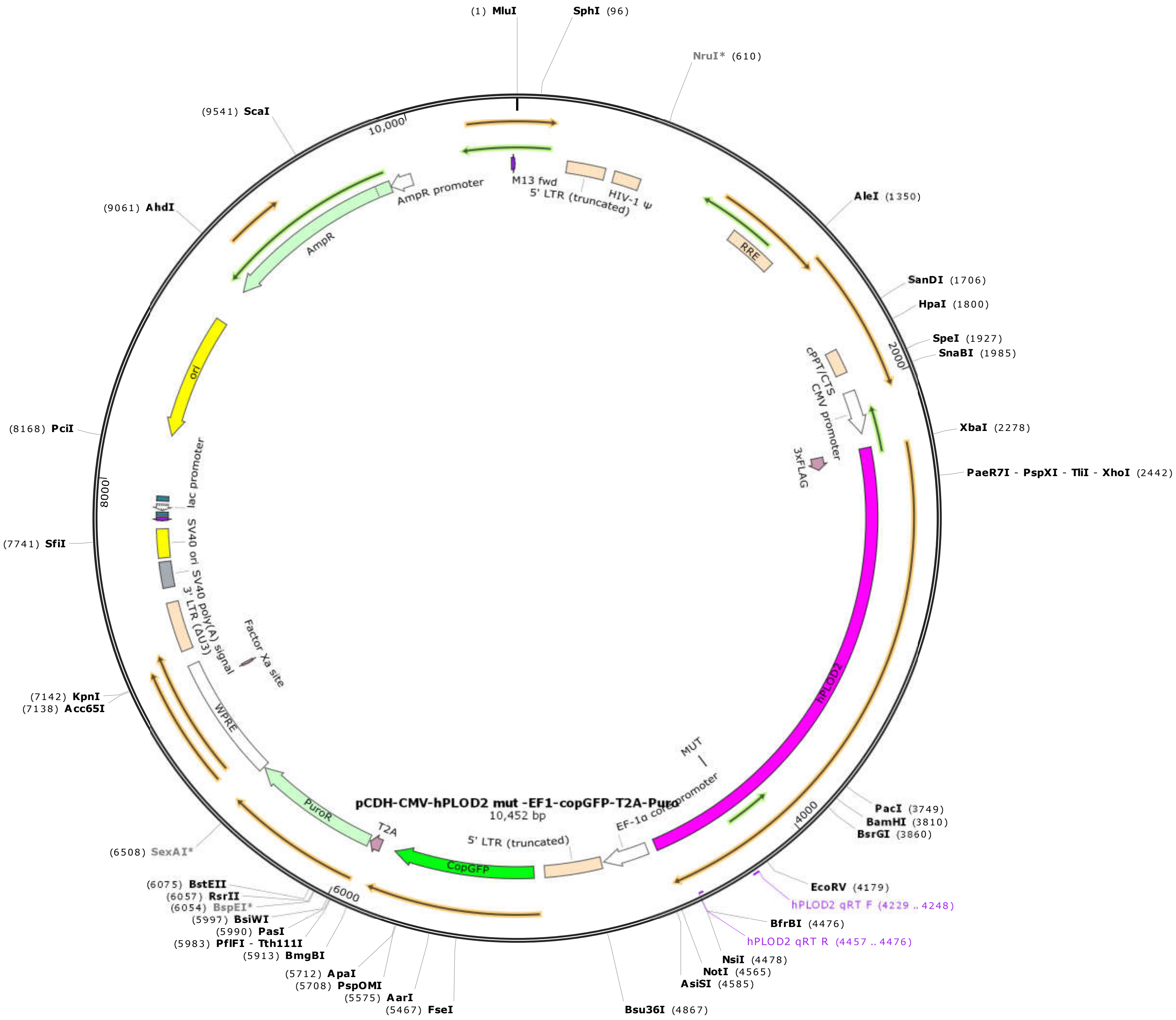
pCDH-CMV-hPLOD2 mut -EF1-copGFP-T2A-Puro
10,452 bp
(1) MluI
SphI (96)
NruI* (610)
AleI (1350)
SanDI (1706)
HpaI (1800)
SpeI (1927)
SnaBI (1985)
XbaI (2278)
PaeR7I - PspXI - TliI - XhoI (2442)
PacI (3749)
BamHI (3810)
BsrGI (3860)
EcoRV (4179)
hPLOD2 qRT F (4229 .. 4248)
BfrBI (4476)
hPLOD2 qRT R (4457 .. 4476)
NsiI (4478)
NotI (4565)
AsiSI (4585)
Bsu36I (4867)
(5467) FseI
(5575) AarI
(5708) PspOMI
(5712) ApaI
(5913) BmgBI
(5983) PflFI - Tth111I
(5990) PasI
(5997) BsiWI
(6054) BspEI*
(6057) RsrII
(6075) BstEII
(6508) SexAI*
(7138) Acc65I
(7142) KpnI
(7741) SfiI
(8168) PciI
(9061) AhdI
(9541) ScaI
10,000
2000
4000
6000
8000
M13 fwd
5' LTR (truncated)
HIV-1 ψ
RRE
cPPT/CTS
CMV promoter
3xFLAG
hPLOD2
MUT
EF-1α core promoter
5' LTR (truncated)
CopGFP
T2A
PuroR
WPRE
Factor Xa site
3' LTR (ΔU3)
SV40 poly(A) signal
SV40 ori
lac promoter
ori
AmpR
AmpR promoter

```
MluI                                                                                                        SphI
5' acgcgtgtagtcttatgcaatactcttgtagtcttgcaacatggtaacgatgagttagcaacatgccttacaaggagagaaaaagcaccgtgcatgccgattggtggaagtaagg 115
3' tgcgcacatcagaatacgttatgagaacatcagaacgttgtaccattgctactcaatcgttgtacggaatgttcctctctttttcgtggcacgtacggctaaccaccttcattcc
   T R V V L C N T L V V L Q H G N D E L A T C L T R R E K A P C M P I G G S K →
←   R T T K H L V R T T K C C P L S S N A V H R V L L S F A G H M G I P P L L T

tggtacgatcgtgccttattaggaaggcaacagacgggtctgacatggattggacgaaccactgaattgccgcattgcagagatattgtatttaagtgcctagctcgatacaata 230
accatgctagcacggaataatccttccgttgtctgcccagactgtacctaacctgcttggtgacttaacggcgtaacgtctctataacataaattcacggatcgagctatgttat
V V R S C L I R K A T D G S D M D W T N H * →
←  T R D H R I L F A V S P D S M

aacgggtctctctggttagaccagatctgagcctgggagctctctggctaactagggaacccactgcttaagcctcaataaagcttgccttgagtgcttcaagtagtgtgtgccc 345
ttgcccagagagaccaatctggtctagactcggaccctcgagagaccgattgatcccttgggtgacgaattcggagttatttcgaacggaactcacgaagttcatcacacacggg
5' LTR (truncated)

gtctgttgtgtgactctggtaactagagatccctcagacccttttagtcagtgtggaaaatctctagcagtggcgcccgaacagggacctgaaagcgaaagggaaaccagagctc 460
cagacaacacactgagaccattgatctctagggagtctgggaaaatcagtcacaccttttagagatcgtcaccgcgggcttgtccctggactttcgctttccctttggtctcgag
5' LTR (truncated)                                                                                             HIV-1 Ψ

tctcgacgcaggactcggcttgctgaagcgcgcacggcaagaggcgaggggcggcgactggtgagtacgccaaaaatttgactagcggaggctagaaggagagagatgggtgcg 575
agagctgcgtcctgagccgaacgacttcgcgcgtgccgttctccgctccccgccgctgaccactcatgcggtttttaaactgatcgcctccgatcttcctctctctacccacgc
HIV-1 Ψ

                                  NruI*
agagcgtcagtattaagcgggggagaattagatcgcgatgggaaaaaattcggttaaggccagggggaaagaaaaaatataaattaaaacatatagtatgggcaagcagggagct 690
tctcgcagtcataattcgccccctcttaatctagcgctacccttttttaagccaattccggtccccctttcttttttatatttaattttgtatatcatacccgttcgtccctcga
HIV-1 Ψ

agaacgattcgcagttaatcctggcctgttagaaacatcagaaggctgtagacaaatactgggacagctacaaccatcccttcagacaggatcagaagaacttagatcattatat 805
tcttgctaagcgtcaattaggaccggacaatctttgtagtcttccgacatctgtttatgaccctgtcgatgttggtagggaagtctgtcctagtcttcttgaatctagtaatata

aatacagtagcaaccctctattgtgtgcatcaaaggatagagataaaagacaccaaggaagctttagacaagatagaggaagagcaaaacaaaagtaagaccaccgcacagcaag 920
ttatgtcatcgttgggagataacacacgtagtttcctatctctattttctgtggttccttcgaaatctgttctatctccttctcgttttgttttcattctggtggcgtgtcgttc
←                                                                   * V L Y L F L L V F T L G G C L L

cggccactgatcttcagacctggaggaggagatatgagggacaattggagaagtgaattatataaatataaagtagtaaaaattgaaccattaggagtagcacccaccaaggcaa 1035
gccggtgactagaagtctggacctcctcctctatactccctgttaacctcttcacttaatatatttatatttcatcatttttaacttggtaatcctcatcgtgggtggttccgtt
                                    M R D N W R S E L Y K Y K V V K I E P L G V A P T K A →
← R G S I K L G P P P S I L S L Q L L S N Y L Y L T T F I S G N P T A G V L A F

agagaagagtggtgcagagagaaaaaagagcagtgggaataggagctttgttccttgggttcttgggagcagcaggaagcactatgggcgcagcctcaatgacgctgacggtaca 1150
tctcttctcaccacgtctctcttttttctcgtcacccttatcctcgaaacaaggaacccaagaaccctcgtcgtccttcgtgatacccgcgtcggagttactgcgactgccatgt
                                          RRE
K R R V V Q R E K R A V G I G A L F L G F L G A A G S T M G A A S M T L T V Q →
←  L L T T C L S F L A T P I P A K N R P N K P A A P L V I P A A E I V S V T C

ggccagacaattattgtctggtatagtgcagcagcagaacaatttgctgagggctattgaggcgcaacagcatctgttgcaactcacagtctggggcatcaagcagctccaggca 1265
ccggtctgttaataacagaccatatcacgtcgtcgtcttgttaaacgactcccgataactccgcgttgtcgtagacaacgttgagtgtcagaccccgtagttcgtcgaggtccgt
RRE
 A R Q L L S G I V Q Q Q N N L L R A I E A Q Q H L L Q L T V W G I K Q L Q A →
← A L C N N D P I T C C C F L K S L A I S A C C C R N C S V T Q P M

                                                                                    AleI
agaatcctggctgtggaaagatacctaaaggatcaacagctcctggggatttggggttgctctggaaaactcatttgcaccactgctgtgccttggaatgctagttggagtaata 1380
tcttaggaccgacacctttctatggatttcctagttgtcgaggacccctaaaccccaacgagaccttttgagtaaacgtggtgacgacacggaaccttacgatcaacctcattat
RRE
R I L A V E R Y L K D Q Q L L G I W G C S G K L I C T T A V P W N A S W S N →

aatctctggaacagattggaatcacacgacctggatggagtgggacagagaaattaacaattacacaagcttaatacactccttaattgaagaatcgcaaaaccagcaagaaaag 1495
ttagagaccttgtctaaccttagtgtgctggacctacctcaccctgtctctttaattgttaatgtgttcgaattatgtgaggaattaacttcttagcgttttggtcgttcttttc
                                    M E W D R E I N N Y T S L I H S L I E E S Q N Q Q E K →
K S L E Q I G I T R P G W S G T E K L T I T Q A * →

aatgaacaagaattattggaattagataaatgggcaagtttgtggaattggtttaacataacaaattggctgtggtatataaaattattcataatgatagtaggaggcttggtag 1610
ttacttgttcttaataaccttaatctatttacccgttcaaacaccttaaccaaattgtattgtttaaccgacaccatatattttaataagtattactatcatcctccgaaccatc
N E Q E L L E L D K W A S L W N W F N I T N W L W Y I K L F I M I V G G L V →

                                                                                                        SanDI
gtttaagaatagttttttgctgtactttctatagtgaatagagttaggcagggatattcaccattatcgtttcagacccacctcccaaccccgaggggacccgacaggcccgaagg 1725
caaattcttatcaaaaacgacatgaaagatatcacttatctcaatccgtccctataagtggtaatagcaaagtctgggtggagggttggggctcccctgggctgtccgggcttcc
G L R I V F A V L S I V N R V R Q G Y S P L S F Q T H L P T P R G P D R P E G →
```

```
                                                                                      HpaI
aatagaagaagaaggtggagagagagacagagacagatccattcgattagtgaacggatctcgacggtatcggttaactttaaaagaaaaggggggattggggggtacagtgca  1840
ttatcttcttcttccacctctctctctgtctctgtctaggtaagctaatcacttgcctagagctgccatagccaattgaaaattttcttttcccccctaaccccccatgtcacgt
                                                                                           cPPT/CTS
 I  E  E  E  G  G  E  R  D  R  D  R  S  I  R  L  V  N  G  S  R  R  Y  R  L  T  F  K  R  K  G  G  I  G  G  Y  S  A  →

                                                                                                  SpeI
ggggaaagaatagtagacataatagcaacagacatacaaactaaagaattacaaaaacaaattacaaaattcaaaattttatcgatactagtattatgcccagtacatgacctta  1955
cccctttcttatcatctgtattatcgttgtctgtatgtttgatttcttaatgtttttgtttaatgttttaagttttaaaatagctatgatcataatacgggtcatgtactggaat
                                           cPPT/CTS
 G  E  R  I  V  D  I  I  A  T  D  I  Q  T  K  E  L  Q  K  Q  I  T  K  F  K  I  L  S  I  L  V  L  C  P  V  H  D  L  →

                          SnaBI
tgggactttcctacttggcagtacatctacgtattagtcatcgctattaccatggtgatgcggttttggcagtacatcaatgggcgtggatagcggtttgactcacggggatttc  2070
accctgaaaggatgaaccgtcatgtagatgcataatcagtagcgataatggtaccactacgccaaaaccgtcatgtagttacccgcacctatcgccaaactgagtgcccctaaag
                                                                                  CMV promoter >
 M  G  L  S  Y  L  A  V  H  L  R  I  S  H  R  Y  Y  H  G  D  A  V  L  A  V  H  Q  W  A  W  I  A  V  *  →

caagtctccaccccattgacgtcaatgggagtttgttttggcaccaaaatcaacgggactttccaaaatgtcgtaacaactccgccccattgacgcaaatgggcggtaggcgtgt  2185
gttcagaggtggggtaactgcagttaccctcaaacaaaaccgtggttttagttgccctgaaaggttttacagcattgttgaggcggggtaactgcgtttacccgccatccgcaca
                                                 CMV promoter >
←             *  H  S  N  T  K  A  G  F  D  V  P  S  E  L  I  D  Y  C  S  R  G  M  S  A  F  P  R  Y  A  H

                                                                                                XbaI
acggtgggaggtttatataagcagagctcgtttagtgaaccgtcagatcgcctggagacgccatccacgctgttttgacctccatagaagattctagaatgGACTACAAAGACCA  2300
tgccaccctccaaatatattcgtctcgagcaaatcacttggcagtctagcggacctctgcggtaggtgcgacaaaactggaggtatcttctaagatcttacCTGATGTTTCTGGT
                                                                                                    1       5
                                                                                                    D  Y  K  D  H
   CMV promoter >                                                                                   3xFLAG >
                                                                                                  hPLOD2
                                                                                                 M  D  Y  K  D  H  →
←  V  T  P  P  K  Y  L  C  L  E  N  L  S  G  D  S  R  R  S  V  G  D  V  S  N  Q  G  G  Y  F  I  R  S  H  V  V  F  V  M

TGACGGTGATTATAAAGATCATGACATCGATTACAAGGATGACGACGACAAGgggggatgcacggtgaagcctcagctgctgctcctggcgctcgtcctccacccctggaatccc  2415
ACTGCCACTAATATTTCTAGTACTGTAGCTAATGTTCCTACTGCTGCTGTTCccccctacgtgccacttcggagtcgacgacgaggaccgcgagcaggaggtggggaccttaggg
          10             15             20
 D  G  D  Y  K  D  H  D  I  D  Y  K  D  D  D  D  K  G  G  C  T  V  K  P  Q  L  L  L  L  A  L  V  L  H  P  W  N  P
                   3xFLAG                          (in frame with 3xFLAG)
                                             hPLOD2
 D  G  D  Y  K  D  H  D  I  D  Y  K  D  D  D  D  K  G  G  C  T  V  K  P  Q  L  L  L  L  A  L  V  L  H  P  W  N  P  →
←  V  T  I  I  F  I  M

                    TliI
                    XhoI
                    PspXI
                    PaeR7I
tgtctgggtgcggactcggagaagccctcgagcatccccacagataaattattagtcataactgtagcaacaaaagaaagtgatggattccatcgatttatgcagtcagccaaat  2530
acagacccacgcctgagcctcttcgggagctcgtaggggtgtctatttaataatcagtattgacatcgttgttttctttcactacctaaggtagctaaatacgtcagtcggttta
 C  L  G  A  D  S  E  K  P  S  S  I  P  T  D  K  L  L  V  I  T  V  A  T  K  E  S  D  G  F  H  R  F  M  Q  S  A  K
                                   (in frame with 3xFLAG)
                                             hPLOD2
 C  L  G  A  D  S  E  K  P  S  S  I  P  T  D  K  L  L  V  I  T  V  A  T  K  E  S  D  G  F  H  R  F  M  Q  S  A  K  →

atttcaattatactgtgaaggtccttggtcaaggagaagaatggagaggtggtgatggaattaatagtattggagggggccagaaagtgagattaatgaaagaagtcatggaaca  2645
taaagttaatatgacacttccaggaaccagttcctcttcttacctctccaccactaccttaattatcataacctcccccggtctttcactctaattactttcttcagtaccttgt
Y  F  N  Y  T  V  K  V  L  G  Q  G  E  E  W  R  G  G  D  G  I  N  S  I  G  G  G  Q  K  V  R  L  M  K  E  V  M  E  H
                                   (in frame with 3xFLAG)
                                             hPLOD2
Y  F  N  Y  T  V  K  V  L  G  Q  G  E  E  W  R  G  G  D  G  I  N  S  I  G  G  G  Q  K  V  R  L  M  K  E  V  M  E  H  →

ctatgctgatcaagatgatctggttgtcatgtttactgaatgctttgatgtcatatttgctggtggtccagaagaagttctaaaaaaattccaaaaggcaaaccacaaagtggtc  2760
gatacgactagttctactagaccaacagtacaaatgacttacgaaactacagtataaacgaccaccaggtcttcttcaagatttttttaaggttttccgtttggtgtttcaccag
 Y  A  D  Q  D  D  L  V  V  M  F  T  E  C  F  D  V  I  F  A  G  G  P  E  E  V  L  K  K  F  Q  K  A  N  H  K  V  V
                                   (in frame with 3xFLAG)
                                             hPLOD2
 Y  A  D  Q  D  D  L  V  V  M  F  T  E  C  F  D  V  I  F  A  G  G  P  E  E  V  L  K  K  F  Q  K  A  N  H  K  V  V  →

tttgcagcagatggaattttgtggccagataaaagactagcagacaagtatcctgttgtgcacattgggaaacgctatctgaattcaggaggatttattggctatgctccatatg  2875
aaacgtcgtctaccttaaaacaccggtctattttctgatcgtctgttcataggacaacacgtgtaaccctttgcgatagacttaagtcctcctaaataaccgatacgaggtatac
 F  A  A  D  G  I  L  W  P  D  K  R  L  A  D  K  Y  P  V  V  H  I  G  K  R  Y  L  N  S  G  G  F  I  G  Y  A  P  Y
                                   (in frame with 3xFLAG)
                                             hPLOD2
 F  A  A  D  G  I  L  W  P  D  K  R  L  A  D  K  Y  P  V  V  H  I  G  K  R  Y  L  N  S  G  G  F  I  G  Y  A  P  Y  →
```

```
tcaaccgtatagttcaacaatggaatctccaggataatgatgatgatcagctcttttacactaaagtttacattgatccactgaaaagggaagctattaacatcacattggatca   2990
agttggcatatcaagttgttaccttagaggtcctattactactactagtcgagaaaatgtgatttcaaatgtaaactaggtgacttttcccttcgataattgtagtgtaacctagt
V N R I V Q Q W N L Q D N D D D Q L F Y T K V Y I D P L K R E A I N I T L D H
(in frame with 3xFLAG)
hPLOD2
V N R I V Q Q W N L Q D N D D D Q L F Y T K V Y I D P L K R E A I N I T L D H

caaatgcaaaattttccagaccttaaatggagctgtagatgaagttgttttaaaatttgaaaatggcaaagccagagctaagaatacattttatgaaacattaccagtggcaatt   3105
gtttacgttttaaaaggtctggaatttacctcgacatctacttcaacaaaattttaaacttttaccgtttcggtctcgattcttatgtaaaatactttgtaatggtcaccgttaa
K C K I F Q T L N G A V D E V V L K F E N G K A R A K N T F Y E T L P V A I
(in frame with 3xFLAG)
hPLOD2
K C K I F Q T L N G A V D E V V L K F E N G K A R A K N T F Y E T L P V A I

aatggaaatggacccaccaagattctcctgaattattttggaaactatgtacccaattcatggacacaggataatggctgcactctttgtgaattcgatacagtcgacttgtctg   3220
ttacctttacctgggtggttctaagaggacttaataaaacctttgatacatgggttaagtacctgtgtcctattaccgacgtgagaaacacttaagctatgtcagctgaacagac
N G N G P T K I L L N Y F G N Y V P N S W T Q D N G C T L C E F D T V D L S
(in frame with 3xFLAG)
hPLOD2
N G N G P T K I L L N Y F G N Y V P N S W T Q D N G C T L C E F D T V D L S

cagtagatgtccatccaaacgtatcaataggtgtttttattgagcaaccaaccccttttctacctcggtttctggacatattgttgacactggattacccaaaagaagcacttaa   3335
gtcatctacaggtaggtttgcatagttatccacaaaaataactcgttggttggggaaaagatggagccaaagacctgtataacaactgtgacctaatgggttttcttcgtgaatt
A V D V H P N V S I G V F I E Q P T P F L P R F L D I L L T L D Y P K E A L K
(in frame with 3xFLAG)
hPLOD2
A V D V H P N V S I G V F I E Q P T P F L P R F L D I L L T L D Y P K E A L K

acttttattcataacaaagaagtttatcatgaaaaggacatcaaggtatttttgataaagctaagcatgaaatcaaaactataaaaatagtaggaccagaagaaaatctaagt   3450
tgaaaaataagtattgtttcttcaaatagtacttttcctgtagttccataaaaaactatttcgattcgtactttagttttgatatttttatcatcctggtcttcttttagattca
L F I H N K E V Y H E K D I K V F F D K A K H E I K T I K I V G P E E N L S
(in frame with 3xFLAG)
hPLOD2
L F I H N K E V Y H E K D I K V F F D K A K H E I K T I K I V G P E E N L S

caagcggaagccagaaacatgggaatggacttttgccgtcaggatgaaaagtgtgattattactttagtgtggatgcagatgttgttttgacaaatccaaggactttaaaaattt   3565
gttcgccttcggtctttgtacccttacctgaaaacggcagtcctacttttcacactaataatgaaatcacacctacgtctacaacaaaactgtttaggttcctgaaatttttaaa
Q A E A R N M G M D F C R Q D E K C D Y Y F S V D A D V V L T N P R T L K I
(in frame with 3xFLAG)
hPLOD2
Q A E A R N M G M D F C R Q D E K C D Y Y F S V D A D V V L T N P R T L K I

tgattgaacaaaacagaaagatcattgctcctcttgtaactcgtcatggaaagctgtggtccaatttctggggagcattgagtcctgatggatactatgcacgatctgaagatta   3680
actaacttgttttgtctttctagtaacgaggagaacattgagcagtacctttcgacaccaggttaaagacccctcgtaactcaggactacctatgatacgtgctagacttctaat
L I E Q N R K I I A P L V T R H G K L W S N F W G A L S P D G Y Y A R S E D Y
(in frame with 3xFLAG)
hPLOD2
L I E Q N R K I I A P L V T R H G K L W S N F W G A L S P D G Y Y A R S E D Y

PacI
tgtggatattgttcaagggaatagagtaggagtatggaatgtcccatatatggctaatgtgtacttaattaaaggaaagacactccgatcagagatgaatgaaaggaactatttt   3795
acacctataacaagttcccttatctcatcctcataccttacagggtatataccgattacacatgaattaatttcctttctgtgaggctagtctctacttactttccttgataaaa
V D I V Q G N R V G V W N V P Y M A N V Y L I K G K T L R S E M N E R N Y F
(in frame with 3xFLAG)
hPLOD2
V D I V Q G N R V G V W N V P Y M A N V Y L I K G K T L R S E M N E R N Y F

BamHI                                        BsrGI
gttcgtgataaactggatcctgatatggctctttgccgaaatgctagagaaatgggtgtatttatgtacatttctaatagacatgaatttggaaggctattatccactgctaatt   3910
caagcactatttgacctaggactataccgagaaacggctttacgatctctttacccacataaatacatgtaaagattatctgtacttaaaccttccgataataggtgacgattaa
V R D K L D P D M A L C R N A R E M G V F M Y I S N R H E F G R L L S T A N
(in frame with 3xFLAG)
hPLOD2
V R D K L D P D M A L C R N A R E M G V F M Y I S N R H E F G R L L S T A N

acaatacttcccattataacaatgacctctggcagattttgaaaatcctgtggactggaaggaaaagtatataaaccgtgattattcaaagattttcactgaaaatatagttga   4025
tgttatgaagggtaatattgttactggagaccgtctaaaactttaggacacctgaccttccttttcatatatttggcactaataagtttctaaaagtgacttttatatcaact
Y N T S H Y N N D L W Q I F E N P V D W K E K Y I N R D Y S K I F T E N I V E
(in frame with 3xFLAG)
hPLOD2
Y N T S H Y N N D L W Q I F E N P V D W K E K Y I N R D Y S K I F T E N I V E
* L N E S F I Y N F
```

acagccctgtccagatgtcttttggttccccatatttctgaaaaagcctgtgatgaattggtagaagaaatggaacattacggcaaatggtctgggggaaaacatcatgatagc 4140
tgtcgggacaggtctacagaaaaccaaggggtataaaagactttttcggacactacttaaccatcttctttaccttgtaatgccgtttaccagacccccttttgtagtactatcg
Q P C P D V F W F P I F S E K A C D E L V E E M E H Y G K W S G G K H H D S
(in frame with 3xFLAG)
hPLOD2
Q P C P D V F W F P I F S E K A C D E L V E E M E H Y G K W S G G K H H D S
L G T W I D K P E G Y K R F F G T I F Q Y F F H F M V A F P R P S F M M I A

EcoRV
hPLOD2 qRT F
gggagttcattgcaccagtt
cAtatatctggtggttatgaaaatgtcccaactgatgatatccacatgaagcaagttgatctggagaatgtatggcttcattttatccgggagttcattgcaccagttacactga 4255
gTatatagaccaccaatacttttacagggttgactactataggtgtacttcgttcaactagacctcttacataccgaagtaaaataggccctcaagtaacgtggtcaatgtgact
H I S G G Y E N V P T D D I H M K Q V D L E N V W L H F I R E F I A P V T L
(in frame with 3xFLAG)
hPLOD2
MUT
H I S G G Y E N V P T D D I H M K Q V D L E N V W L H F I R E F I A P V T L
M Y R T T I F I D W S I I D V H L L N I Q L I Y P K M

aggtctttgcaggctattatacgaagggatttgcactactgaatttttgtagtaaaatactcccctgaacgacagcgttctcttcgtcctcatcatgatgcttctacatttaccat 4370
tccagaaacgtccgataatatgcttccctaaacgtgatgacttaaaaacatcatttatgaggggacttgctgtcgcaagagaagcaggagtagtactacgaagatgtaaatggta
K V F A G Y Y T K G F A L L N F V V K Y S P E R Q R S L R P H H D A S T F T I
(in frame with 3xFLAG)
hPLOD2
K V F A G Y Y T K G F A L L N F V V K Y S P E R Q R S L R P H H D A S T F T I

BfrBI NsiI
aaacattgcacttaataacgtgggagaagactttcagggaggtggttgcaaatttctaaggtacaattgctctattgagtcaccacgaaaaggctggagcttcatgcatcctggg 4485
tttgtaacgtgaattattgcaccctcttctgaaagtccctccaccaacgtttaaagattccatgttaacgagataactcagtggtgcttttccgacctcgaagtacgtaggaccc
N I A L N N V G E D F Q G G G C K F L R Y N C S I E S P R K G W S F M H P G
(in frame with 3xFLAG)
hPLOD2
N I A L N N V G E D F Q G G G C K F L R Y N C S I E S P R K G W S F M H P G
cttttccgacctcgaagtac
hPLOD2 qRT R

NotI AsiSI
agactcacacatttgcatgaaggacttcctgttaaaaatggaacaagatacattgcagtgtcatttatagatccctaagcggccgcgaaggatctgcgatcgctccggtgcccgt 4600
tctgagtgtgtaaacgtacttcctgaaggacaatttttaccttgttctatgtaacgtcacagtaaatatctagggattcgccggcgcttcctagacgctagcgaggccacgggca
R L T H L H E G L P V K N G T R Y I A V S F I D P
(in frame with 3xFLAG)
hPLOD2
R L T H L H E G L P V K N G T R Y I A V S F I D P

cagtgggcagagcgcacatcgcccacagtccccgagaagttggggggaggggtcggcaattgaacgggtgcctagagaaggtggcgcggggtaaactgggaaagtgatgtcgtgt 4715
gtcacccgtctcgcgtgtagcgggtgtcaggggctcttcaaccccccctccccagccgttaacttgcccacggatctcttccaccgcgccccatttgaccctttcactacagcaca
EF-1α core promoter

actggctccgcctttttcccgagggtgggggagaaccgtatataagtgcagtagtcgccgtgaacgttctttttcgcaacgggtttgccgccagaacacagctgaagcttcgagg 4830
tgaccgaggcggaaaaagggctcccacccccctcttggcatatattcacgtcatcagcggcacttgcaagaaaaagcgttgcccaaacggcggtcttgtgtcgacttcgaagctcc
EF-1α core promoter
5' LTR (truncated)

Bsu36I
ggctcgcatctctccttcacgcgcccgccgccctacctgaggccgccatccacgccggttgagtcgcgttctgccgcctcccgcctgtggtgcctcctgaactgcgtccgccgtc 4945
ccgagcgtagagaggaagtgcgcgggcggcgggatggactccggcggtaggtgcggccaactcagcgcaagacggcggagggcggacaccacggaggacttgacgcaggcggcag
5' LTR (truncated)

taggtaagtttaaagctcaggtcgagaccgggcctttgtccggcgctcccttggagcctacctagactcagccggctctccacgctttgcctgaccctgcttgctcaactctacg 5060
atccattcaaatttcgagtccagctctggcccggaaacaggccgcgagggaacctcggatggatctgagtcggccgagaggtgcgaaacggactgggacgaacgagttgagatgc
5' LTR (truncated)

tctttgtttcgttttctgttctgcgccgttacagatccaagctgtgaccggcgcctacgctagatggagagcgacgagagcggcctgcccgccatggagatcgagtgccgcatca 5175
agaaacaaagcaaaagacaagacgcggcaatgtctaggttcgacactggccgcggatgcgatctacctctcgctgctctcgccggacgggcggtacctctagctcacggcgtagt
5 10
P A M E I E C R I
5' LTR (truncated)
CopGFP
M E S D E S G L P A M E I E C R I

ccggcaccctgaacggcgtggagttcgagctggtgggcggcggagagggcacccccaagcagggccgcatgaccaacaagatgaagagcaccaaaggcgccctgaccttcagccc 5290
ggccgtgggacttgccgcacctcaagctcgaccacccgccgcctctcccgtggggttcgtcccggcgtactggttgttctacttctcgtggtttccgcgggactggaagtcggg
15 20 25 30 35 40 45
T G T L N G V E F E L V G G G E G T P K Q G R M T N K M K S T K G A L T F S P
CopGFP
T G T L N G V E F E L V G G G E G T P K Q G R M T N K M K S T K G A L T F S P

```
ctacctgctgagccacgtgatgggctacggcttctaccacttcggcacctaccccagcggctacgagaaccccttcctgcacgccatcaacaacggcggctacaccaacacccgc   5405
gatggacgactcggtgcactacccgatgccgaagatggtgaagccgtggatggggtcgccgatgctcttggggaaggacgtgcggtagttgttgccgccgatgtggttgtgggcg
 50    55    60    65    70    75    80    85
 Y L L S H V M G Y G F Y H F G T Y P S G Y E N P F L H A I N N G G Y T N T R
 CopGFP
 Y L L S H V M G Y G F Y H F G T Y P S G Y E N P F L H A I N N G G Y T N T R

FseI
atcgagaagtacgaggacggcggcgtgctgcacgtgagcttcagctaccgctacgaggccggccgcgtgatcggcgacttcaaggtggtgggcaccggcttccccgaggacagcg   5520
tagctcttcatgctcctgccgccgcacgacgtgcactcgaagtcgatggcgatgctccggccggcgcactagccgctgaagttccaccacccgtggccgaaggggctcctgtcgc
 90    95    100    105    110    115    120    125
 I E K Y E D G G V L H V S F S Y R Y E A G R V I G D F K V V G T G F P E D S
 CopGFP
 I E K Y E D G G V L H V S F S Y R Y E A G R V I G D F K V V G T G F P E D S

AarI
tgatcttcaccgacaagatcatccgcagcaacgccaccgtggagcacctgcaccccatgggcgataacgtgctggtgggcagcttcgcccgcaccttcagcctgcgcgacggcgg   5635
actagaagtggctgttctagtaggcgtcgttgcggtggcacctcgtggacgtggggtacccgctattgcacgaccacccgtcgaagcgggcgtggaagtcggacgcgctgccgcc
 130    135    140    145    150    155    160
V I F T D K I I R S N A T V E H L H P M G D N V L V G S F A R T F S L R D G G
 CopGFP
V I F T D K I I R S N A T V E H L H P M G D N V L V G S F A R T F S L R D G G

PspOMI  ApaI
ctactacagcttcgtggtggacagccacatgcacttcaagagcgccatccaccccagcatcctgcagaacggggggcccatgttcgccttccgccgcgtggaggagctgcacagc   5750
gatgatgtcgaagcaccacctgtcggtgtacgtgaagttctcgcggtaggtggggtcgtaggacgtcttgccccccgggtacaagcggaaggcggcgcacctcctcgacgtgtcg
165    170    175    180    185    190    195    200
 Y Y S F V V D S H M H F K S A I H P S I L Q N G G P M F A F R R V E E L H S
 CopGFP
 Y Y S F V V D S H M H F K S A I H P S I L Q N G G P M F A F R R V E E L H S

aacaccgagctgggcatcgtggagtaccagcacgccttcaagacccccatcgccttcgccagatcccgcgctcagtcgtccaattctgccgtggacggcaccgccggacccggct   5865
ttgtggctcgacccgtagcacctcatggtcgtgcggaagttctgggggtagcggaagcggtctagggcgcgagtcagcaggttaagacggcacctgccgtggcggcctgggccga
 205    210    215    220
N T E L G I V E Y Q H A F K T P I A F A R S R A Q S S N S A V D G T A G P G
 CopGFP    (in frame with CopGFP)
N T E L G I V E Y Q H A F K T P I A F A R S R A Q S S N S A V D G T A G P G

BmgBI
ccaccggatctcgctgagggcagaggaagtcttctaacatgcggtgacgtggaggagaatcccggcccttccgggatgaccgagtacaagcccacggtgcgcctcgccacccgcg   5980
ggtggcctagagcgactcccgtctccttcagaagattgtacgccactgcacctcctcttagggccgggaaggccctactggctcatgttcgggtgccacgcggagcggtgggcgc
S T G S R ■    1    5    10
               M T E Y K P T V R L A T R
               PuroR
 140    145    150    155
 E G R G S L L T C G D V E E N P G P S G
 T2A
               M T E Y K P T V R L A T R
S T G S R ■

PflFI
Tth111I  PasI  BsiWI    BspEI*  RsrII  BstEII
acgacgtccccagggccgtacgcaccctcgccgccgcgttcgccgactaccccgccacgcgccacaccgtcgatccggaccgccacatcgagcgggtcaccgagctgcaagaact   6095
tgctgcaggggtcccggcatgcgtgggagcggcggcgcaagcggctgatggggcggtgcgcggtgtggcagctaggcctggcggtgtagctcgcccagtggctcgacgttcttga
 15    20    25    30    35    40    45    50
D D V P R A V R T L A A A F A D Y P A T R H T V D P D R H I E R V T E L Q E L
 PuroR
D D V P R A V R T L A A A F A D Y P A T R H T V D P D R H I E R V T E L Q E L

cttcctcacgcgcgtcgggctcgacatcggcaaggtgtgggtcgcggacgacggcgccgcggtggcggtctggaccacgccggagagcgtcgaagcgggggcggtgttcgccgag   6210
gaaggagtgcgcgcagcccgagctgtagccgttccacacccagcgcctgctgccgcggcgccaccgccagacctggtgcggcctctcgcagcttcgcccccgccacaagcggctc
 55    60    65    70    75    80    85    90
 F L T R V G L D I G K V W V A D D G A A V A V W T T P E S V E A G A V F A E
 PuroR
 F L T R V G L D I G K V W V A D D G A A V A V W T T P E S V E A G A V F A E

atcggcccgcgcatggccgagttgagcggttcccggctggccgcgcagcaacagatggaaggcctcctggcgccgcaccggcccaaggagcccgcgtggttcctggccaccgtcg   6325
tagccgggcgcgtaccggctcaactcgccaagggccgaccggcgcgtcgttgtctaccttccggaggaccgcggcgtggccgggttcctcgggcgcaccaaggaccggtggcagc
 95    100    105    110    115    120    125
 I G P R M A E L S G S R L A A Q Q Q M E G L L A P H R P K E P A W F L A T V
 PuroR
 I G P R M A E L S G S R L A A Q Q Q M E G L L A P H R P K E P A W F L A T V

gcgtctcgcccgaccaccagggcaagggtctgggcagcgccgtcgtgctccccggagtggaggcggccgagcgcgccggggtgcccgccttcctggagacctccgcgccccgcaa   6440
cgcagagcgggctggtggtcccgttcccagacccgtcgcggcagcacgaggggcctcacctccgccggctcgcgcggccccacgggcggaaggacctctggaggcgcggggcgtt
 130    135    140    145    150    155    160    165
G V S P D H Q G K G L G S A V V L P G V E A A E R A G V P A F L E T S A P R N
 PuroR
G V S P D H Q G K G L G S A V V L P G V E A A E R A G V P A F L E T S A P R N
```

```
SexAI*
cctccccttctacgagcggctcggcttcaccgtcaccgccgacgtcgaggtgcccgaaggaccgcgcacctggtgcatgacccgcaagcccggtgcctgagtcgacaatcaacct   6555
ggaggggaagatgctcgccgagccgaagtggcagtggcggctgcagctccacgggcttcctggcgcgtggaccacgtactgggcgttcgggccacggactcagctgttagttgga
   170   175   180   185   190   195   200
L P F Y E R L G F T V T A D V E V P E G P R T W C M T R K P G A *
PuroR                                  WPRE
L P F Y E R L G F T V T A D V E V P E G P R T W C M T R K P G A *
```

```
ctggattacaaaatttgtgaaagattgactggtattcttaactatgttgctccttttacgctatgtggatacgctgctttaatgcctttgtatcatgctattgcttcccgtatgg   6670
gacctaatgttttaaacactttctaactgaccataagaattgatacaacgaggaaaatgcgatacacctatgcgacgaaattacggaaacatagtacgataacgaagggcatacc
WPRE
M P L Y H A I A S R M
M L L L P V W
```

```
ctttcatttctcctccttgtataaatcctggttgctgtctctttatgaggagttgtggcccgttgtcaggcaacgtggcgtggtgtgcactgtgtttgctgacgcaacccccac   6785
gaaagtaaaagaggaggaacatatttaggaccaacgacagagaaatactcctcaacaccgggcaacagtccgttgcaccgcaccacacgtgacacaaacgactgcgttgggggtg
WPRE
A F I F S S L Y K S W L L S L Y E E L W P V V R Q R G V V C T V F A D A T P T
L S F S P P C I N P G C C L F M R S C G P L S G N V A W C A L C L L T Q P P
```

```
tggttggggcattgccaccacctgtcagctcctttccgggactttcgctttccccctccctattgccacggcggaactcatcgccgcctgccttgcccgctgctggacaggggct   6900
accaaccccgtaacggtggtggacagtcgaggaaaggccctgaaagcgaaagggggagggataacggtgccgccttgagtagcggcggacggaacgggcgacgacctgtccccga
WPRE
G W G I A T T C Q L L S G T F A F P L P I A T A E L I A A C L A R C W T G A
L V G A L P P P V S S F P G L S L S P S L L P R R N S S P P A L P A A G Q G L
```

```
cggctgttgggcactgacaattccgtggtgttgtcggggaaatcatcgtcctttccttggctgctcgcctgtgttgccacctggattctgcgcgggacgtccttctgctacgtcc   7015
gccgacaacccgtgactgttaaggcaccacaacagcccctttagtagcaggaaaggaaccgacgagcggacacaacggtggacctaagacgcgccctgcaggaagacgatgcagg
* R G K R P Q E G T N G G P N Q A P R G E A V D
(in frame with Factor Xa site)
WPRE
R L L G T D N S V V L S G K S S S F P W L L A C V A T W I L R G T S F C Y V
G C W A L T I P W C C R G N H R P F L G C S P V L P P G F C A G R P S A T S
```

```
cttcggccctcaatccagcggaccttccttcccgcggcctgctgccggctctgcggcctcttccgcgtcttcgccttcgccctcagacgagtcggatctccctttgggccgcctc   7130
gaagccgggagttaggtcgcctggaaggaagggcgccggacgacggccgagacgccggagaaggcgcagaagcggaagcgggagtctgctcagcctagagggaaacccggcggag
     1
R R G E I
Factor Xa site
WPRE
P S A L N P A D L P S R G L L P A L R P L P R L R L R P Q T S R I S L W A A S
L R P S I Q R T F L P A A C C R L C G L F R V F A F A L R R V G S P F G P P
```

```
Acc65I   KpnI
cccgcctggtacctttaagaccaatgacttacaaggcagctgtagatcttagccactttttaaaagaaaaggggggactggaagggctaattcactcccaacgaaaataagatct   7245
gggcggaccatggaaattctggttactgaatgttccgtcgacatctagaatcggtgaaaaattttcttttcccccctgaccttcccgattaagtgagggttgcttttattctaga
WPRE                                                   3' LTR (ΔU3)
P P G T F K T N D L Q G S C R S *
P R L V P L R P M T Y K A A V D L S H F L K E K G G L E G L I H S Q R K *
```

```
gctttttgcttgtactgggtctctctggttagaccagatctgagcctgggagctctctggctaactagggaacccactgcttaagcctcaataaagcttgccttgagtgcttcaa   7360
cgaaaaacgaacatgacccagagagaccaatctggtctagactcggaccctcgagagaccgattgatcccttgggtgacgaattcggagttatttcgaacggaactcacgaagtt
3' LTR (ΔU3)
```

```
gtagtgtgtgcccgtctgttgtgtgactctggtaactagagatccctcagacccttttagtcagtgtggaaaatctctagcagtagtagttcatgtcatcttattattcagtatt   7475
catcacacacgggcagacaacacactgagaccattgatctctagggagtctgggaaaatcagtcacaccttttagagatcgtcatcatcaagtacagtagaataataagtcataa
3' LTR (ΔU3)
```

```
tataacttgcaaagaaatgaatatcagagagtgagaggaacttgtttattgcagcttataatggttacaaataaagcaatagcatcacaaatttcacaaataaagcatttttttc   7590
atattgaacgtttctttacttatagtctctcactctccttgaacaaataacgtcgaatattaccaatgtttatttcgttatcgtagtgtttaaagtgtttatttcgtaaaaaaag
SV40 poly(A) signal
```

```
actgcattctagttgtggtttgtccaaactcatcaatgtatcttatcatgtctggctctagctatcccgcccctaactccgcccagttccgcccattctccgccccatggctgac   7705
tgacgtaagatcaacaccaaacaggtttgagtagttacatagaatagtacagaccgagatcgatagggcggggattgaggcgggtcaaggcgggtaagaggcggggtaccgactg
SV40 poly(A) signal                          SV40 ori
```

```
SfiI
taatttttttatttatgcagaggccgaggccgcctcggcctctgagctattccagaagtagtgaggaggcttttttggaggcctagacttttgcagagacggcccaaattcgta   7820
attaaaaaaataaatacgtctccggctccggcggagccggagactcgataaggtcttcatcactcctccgaaaaaacctccggatctgaaaacgtctctgccgggtttaagcat
SV40 ori
```

```
atcatggtcatagctgtttcctgtgtgaaattgttatccgctcacaattccacacaacatacgagccggaagcataaagtgtaaagcctggggtgcctaatgagtgagctaactc   7935
tagtaccagtatcgacaaaggacacactttaacaataggcgagtgttaaggtgtgttgtatgctcggccttcgtatttcacatttcggaccccacggattactcactcgattgag
M13 rev   lac operator   -10   -35   CAP binding site
lac promoter
```

acattaattgcgttgcgctcactgcccgctttccagtcgggaaacctgtcgtgccagctgcattaatgaatcggccaacgcgcggggagaggcggtttgcgtattgggcgctctt 8050
tgtaattaacgcaacgcgagtgacgggcgaaaggtcagccctttggacagcacggtcgacgtaattacttagccggttgcgcgcccctctccgccaaacgcataacccgcgagaa

CAP binding site

ccgcttcctcgctcactgactcgctgcgctcggtcgttcggctgcggcgagcggtatcagctcactcaaaggcggtaatacggttatccacagaatcaggggataacgcaggaaa 8165
ggcgaaggagcgagtgactgagcgacgcgagccagcaagccgacgccgctcgccatagtcgagtgagtttccgccattatgccaataggtgtcttagtcccctattgcgtccttt

PciI

gaacatgtgagcaaaaggccagcaaaaggccaggaaccgtaaaaaggccgcgttgctggcgtttttccataggctccgcccccctgacgagcatcacaaaaatcgacgctcaagt 8280
cttgtacactcgttttccggtcgttttccggtccttggcatttttccggcgcaacgaccgcaaaaaggtatccgaggcggggggactgctcgtagtgtttttagctgcgagttca

ori

cagaggtggcgaaacccgacaggactataaagataccaggcgtttccccctggaagctccctcgtgcgctctcctgttccgaccctgccgcttaccggatacctgtccgcctttc 8395
gtctccaccgctttgggctgtcctgatatttctatggtccgcaaagggggaccttcgagggagcacgcgagaggacaaggctgggacggcgaatggcctatggacaggcggaaag

ori

tcccttcgggaagcgtggcgctttctcatagctcacgctgtaggtatctcagttcggtgtaggtcgttcgctccaagctgggctgtgtgcacgaaccccccgttcagcccgaccg 8510
agggaagcccttcgcaccgcgaaagagtatcgagtgcgacatccatagagtcaagccacatccagcaagcgaggttcgacccgacacacgtgcttggggggcaagtcgggctggc

ori

ctgcgccttatccggtaactatcgtcttgagtccaacccggtaagacacgacttatcgccactggcagcagccactggtaacaggattagcagagcgaggtatgtaggcggtgct 8625
gacgcggaataggccattgatagcagaactcaggttgggccattctgtgctgaatagcggtgaccgtcgtcggtgaccattgtcctaatcgtctcgctccatacatccgccacga

ori

acagagttcttgaagtggtggcctaactacggctacactagaaggacagtatttggtatctgcgctctgctgaagccagttaccttcggaaaaagagttggtagctcttgatccg 8740
tgtctcaagaacttcaccaccggattgatgccgatgtgatcttcctgtcataaaccatagacgcgagacgacttcggtcaatggaagcctttttctcaaccatcgagaactaggc

ori

gcaaacaaaccaccgctggtagcggtggtttttttgtttgcaagcagcagattacgcgcagaaaaaaaggatctcaagaagatcctttgatcttttctacggggtctgacgctca 8855
cgtttgtttggtggcgaccatcgccaccaaaaaaacaaacgttcgtcgtctaatgcgcgtcttttttttcctagagttcttctaggaaactagaaaagatgccccagactgcgagt

ori

gtggaacgaaaactcacgttaagggattttggtcatgagattatcaaaaaggatcttcacctagatccttttaaattaaaaatgaagttttaaatcaatctaaagtatatatgag 8970
caccttgcttttgagtgcaattccctaaaaccagtactctaatagtttttcctagaagtggatctaggaaaatttaatttttacttcaaaatttagttagatttcatatatactc

AhdI

taaacttggtctgacagttaccaatgcttaatcagtgaggcacctatctcagcgatctgtctatttcgttcatccatagttgcctgactccccgtcgtgtagataactacgatac 9085
atttgaaccagactgtcaatggttacgaattagtcactccgtggatagagtcgctagacagataaagcaagtaggtatcaacggactgaggggcagcacatctattgatgctatg

285 280 275 270 265 260 255
* W H K I L S A G I E A I Q R N R E D M T A Q S G T T Y I V V I R

AmpR

* W H K I L S A G I E A I Q R N R E D M T A Q S G T T Y I V V I R

gggagggcttaccatctggccccagtgctgcaatgataccgcgagacccacgctcaccggctccagatttatcagcaataaaccagccagccggaagggccgagcgcagaagtgg 9200
ccctcccgaatggtagaccggggtcacgacgttactatggcgctctgggtgcgagtggccgaggtctaaatagtcgttatttggtcggtcggccttcccggctcgcgtcttcacc

250 245 240 235 230 225 220
S P K G D P G L A A I I G R S G R E G A G S K D A I F W G A P L A S R L L P

AmpR

M I P R D P R S P A P D L S A I N Q P A G R A E R R S G

S P K G D P G L A A I I G R S G R E G A G S K D A I F W G A P L A S R L L P

tcctgcaactttatccgcctccatccagtctattaattgttgccgggaagctagagtaagtagttcgccagttaatagtttgcgcaacgttgttgccattgctacaggcatcgtg 9315
aggacgttgaaataggcggaggtaggtcagataattaacaacggcccttcgatctcattcatcaagcggtcaattatcaaacgcgttgcaacaacggtaacgatgtccgtagcac

215 210 205 200 195 190 185 180
G A V K D A E M W D I L Q Q R S A L T L L E G T L L K R L T T A M A V P M T

AmpR

P A T L S A S I Q S I N C C R E A R V S S S P V N S L R N V V A I A T G I V

G A V K D A E M W D I L Q Q R S A L T L L E G T L L K R L T T A M A V P M T

gtgtcacgctcgtcgtttggtatggcttcattcagctccggttcccaacgatcaaggcgagttacatgatcccccatgttgtgcaaaaaagcggttagctccttcggtcctccga 9430
cacagtgcgagcagcaaaccataccgaagtaagtcgaggccaagggttgctagttccgctcaatgtactagggggtacaacacgttttttcgccaatcgaggaagccaggaggct

175 170 165 160 155 150 145 140
T D R E D N P I A E N L E P E W R D L R T V H D G M N H L F A T L E K P G G I

AmpR

V S R S S F G M A S F S S G S Q R S R R V T *

T D R E D N P I A E N L E P E W R D L R T V H D G M N H L F A T L E K P G G I

ScaI

```
tcgttgtcagaagtaagttggccgcagtgttatcactcatggttatggcagcactgcataattctcttactgtcatgccatccgtaagatgcttttctgtgactggtgagtactc   9545
agcaacagtcttcattcaaccggcgtcacaatagtgagtaccaataccgtcgtgacgtattaagagaatgacagtacggtaggcattctacgaaaagacactgaccactcatgag
    135         130         125         120         115         110         105
T T L L L N A A T N D S M T I A A S C L E R V T M G D T L H K E T V P S Y E
AmpR
T T L L L N A A T N D S M T I A A S C L E R V T M G D T L H K E T V P S Y E

aaccaagtcattctgagaatagtgtatgcggcgaccgagttgctcttgcccggcgtcaatacgggataataccgcgccacatagcagaactttaaaagtgctcatcattggaaaa   9660
ttggttcagtaagactcttatcacatacgccgctggctcaacgagaacgggccgcagttatgccctattatggcgcggtgtatcgtcttgaaattttcacgagtagtaacctttt
  100         95          90          85          80          75          70          65
V L D N Q S Y H I R R G L Q E Q G A D I R S L V A G C L L V K F T S M M P F
AmpR
V L D N Q S Y H I R R G L Q E Q G A D I R S L V A G C L L V K F T S M M P F

cgttcttcggggcgaaaactctcaaggatcttaccgctgttgagatccagttcgatgtaacccactcgtgcacccaactgatcttcagcatcttttactttcaccagcgtttctg   9775
gcaagaagccccgcttttgagagttcctagaatggcgacaactctaggtcaagctacattgggtgagcacgtgggttgactagaagtcgtagaaaatgaaagtggtcgcaaagac
    60          55          50          45          40          35          30          25
R E E P R F S E L I K G S N L D L E I Y G V R A G L Q D E A D K V K V L T E P
AmpR
R E E P R F S E L I K G S N L D L E I Y G V R A G L Q D E A D K V K V L T E P

ggtgagcaaaaacaggaaggcaaaatgccgcaaaaaagggaataagggcgacacggaaatgttgaatactcatactcttcctttttcaatattattgaagcatttatcagggtta   9890
ccactcgtttttgtccttccgttttacggcgttttttcccttattcccgctgtgcctttacaacttatgagtatgagaaggaaaaagttataataacttcgtaaatagtcccaat
      20          15          10          5     1
H A F V P L C F A A F F P I L A V R F H Q I S M
signal sequence                                   AmpR promoter
AmpR
H A F V P L C F A A F F P I L A V R F H Q I S M

ttgtctcatgagcggatacatatttgaatgtatttagaaaaataaacaaataggggttccgcgcacatttccccgaaaagtgccacctgacgtctaagaaaccattattatcatg   10,005
aacagagtactcgcctatgtataaacttacataaatctttttatttgtttatccccaaggcgcgtgtaaaggggcttttcacggtggactgcagattctttggtaataatagtac
AmpR promoter

acattaacctataaaaataggcgtatcacgaggccctttcgtctcgcgcgtttcggtgatgacggtgaaaacctctgacacatgcagctcccggagacggtcacagcttgtctgt   10,120
tgtaattggatatttttatccgcatagtgctccgggaaagcagagcgcgcaaagccactactgccacttttggagactgtgtacgtcgagggcctctgccagtgtcgaacagaca

aagcggatgccgggagcagacaagcccgtcagggcgcgtcagcgggtgttggcgggtgtcggggctggcttaactatgcggcatcagagcagattgtactgagagtgcaccatat   10,235
ttcgcctacggccctcgtctgttcgggcagtcccgcgcagtcgcccacaaccgcccacagccccgaccgaattgatacgccgtagtctcgtctaacatgactctcacgtggtata
M
A A D S C I T S L T C W I

gcggtgtgaaataccgcacagatgcgtaaggagaaaataccgcatcaggcgccattcgccattcaggctgcgcaactgttgggaagggcgatcggtgcgggcctcttcgctatta   10,350
cgccacactttatggcgtgtctacgcattcctcttttatggcgtagtccgcggtaagcggtaagtccgacgcgttgacaacccttcccgctagccacgcccggagaagcgataat
R C E I P H R C V R R K Y R I R R H S P F R L R N C W E G R S V R A S S L L
R H S I G C L H T L L F Y R M L R W E G N L S R L Q Q S P R D T R A E E S N

cgccagctggcgaaagggggatgtgctgcaaggcgattaagttgggtaacgccagggttttcccagtcacgacgttgtaaaacgacggccagtgccaagctg ... 3'   10,452
gcggtcgaccgctttccccctacacgacgttccgctaattcaacccattgcggtcccaaaagggtcagtgctgcaacattttgctgccggtcacggttcgac     5'
M13 fwd
R Q L A K G G C A A R R L S W V T P G F S Q S R R C K T T A S A K L
R W S A F P P H A A L R N L Q T V G P N E W D R R Q L V V A L A L S
```
