# Supplementary material for: Genetic Analysis and Functional Study of a Pedigree With Bruck Syndrome Caused by PLOD2 Variant
Source: Front Pediatr. 2022 May 6;10:878172. doi: 10.3389/fped.2022.878172 (PMC9120662; doi:10.3389/fped.2022.878172)
Supplement: Supplementary Material 3 — The names of the repository/repositories and accession number(s). [file Data_Sheet_3.pdf]

We have uploaded the data to GenBank. The accession number is SCV001837654 (https://submit.ncbi.nlm.nih.gov/subs/clinvar_wizard/SUB10342987/overview). In addition, a screenshot of the data upload is listed below.

Review & Submit

Info

Submission name	SUB10342987
Data from a named study	no
Release status	public
Accessions requested	yes

Organization(s)

Organization(s)	Department of Traditional Chinese Medicine
From a single organization or multiple organizations?	single
On behalf of another organization?	no

Variant

Type	single
Method of description	hgvs
Assembly	GRCh38
Accession and version of reference sequence	NM_182943.3
Description of the sequence change	c.1856G>A
Gene	PLOD2: procollagen-lysine,2-oxoglutarate 5-dioxygenase 2
